# Unique alcohol dehydrogenases involved in algal sugar utilization by marine bacteria

**DOI:** 10.1007/s00253-023-12447-x

**Published:** 2023-03-07

**Authors:** Stefan Brott, Ki Hyun Nam, François Thomas, Theresa Dutschei, Lukas Reisky, Maike Behrens, Hanna C. Grimm, Gurvan Michel, Thomas Schweder, Uwe T. Bornscheuer

**Affiliations:** 1 Department of Biotechnology & Enzyme Catalysis, Institute of Biochemistry, University of Greifswald, 17487 Greifswald, Germany; 2 Department of Life Science, Pohang University of Science and Technology, Pohang, 37673 South Korea; 3 Laboratory of Integrative Biology of Marine Models (LBI2M), Station Biologique de Roscoff (SBR), Sorbonne Université, CNRS 29688, Roscoff, Bretagne France; 4 Department of Pharmaceutical Biotechnology, Institute of Pharmacy, University of Greifswald, 17487 Greifswald, Germany

**Keywords:** Alcohol dehydrogenase, Porphyran, CAZyme, Bacteroidetes, *Zobellia galactanivorans*, Auxiliary activity

## Abstract

**Abstract:**

Marine algae produce complex polysaccharides, which can be degraded by marine heterotrophic bacteria utilizing carbohydrate-active enzymes. The red algal polysaccharide porphyran contains the methoxy sugar 6-*O*-methyl-d-galactose (G6Me). In the degradation of porphyran, oxidative demethylation of this monosaccharide towards d-galactose and formaldehyde occurs, which is catalyzed by a cytochrome P450 monooxygenase and its redox partners. In direct proximity to the genes encoding for the key enzymes of this oxidative demethylation, genes encoding for zinc-dependent alcohol dehydrogenases (ADHs) were identified, which seem to be conserved in porphyran utilizing marine *Flavobacteriia*. Considering the fact that dehydrogenases could play an auxiliary role in carbohydrate degradation, we aimed to elucidate the physiological role of these marine ADHs. Although our results reveal that the ADHs are not involved in formaldehyde detoxification, a knockout of the ADH gene causes a dramatic growth defect of *Zobellia galactanivorans* with G6Me as a substrate. This indicates that the ADH is required for G6Me utilization. Complete biochemical characterizations of the ADHs from *Formosa agariphila* KMM 3901^T^ (FoADH) and *Z. galactanivorans* Dsij^T^ (ZoADH) were performed, and the substrate screening revealed that these enzymes preferentially convert aromatic aldehydes. Additionally, we elucidated the crystal structures of FoADH and ZoADH in complex with NAD^+^ and showed that the strict substrate specificity of these new auxiliary enzymes is based on a narrow active site.

**Key points:**

*• Knockout of the ADH-encoding gene revealed its role in 6-O-methyl-D-galactose utilization, suggesting a new auxiliary activity in marine carbohydrate degradation.*

*• Complete enzyme characterization indicated no function in a subsequent reaction of the oxidative demethylation, such as formaldehyde detoxification.*

*• These marine ADHs preferentially convert aromatic compounds, and their strict substrate specificity is based on a narrow active site.*

**Supplementary Information:**

The online version contains supplementary material available at 10.1007/s00253-023-12447-x.

## Introduction

Marine algae represent one of the most crucial primary producers within the marine carbon cycle and contribute to approximately 50% of the total global primary production (Field 1998). For instance, macroalgae sequester approximately 173 GT of carbon dioxide/year (Krause-Jensen and Duarte 2016) and accumulate the excess carbon in the form of carbohydrates, which they utilize as cell wall constituents or for energy storage (Arnosti et al. 2021). Degradation of these marine polysaccharides can be extremely complicated due to their complexity and the occurrence of side chain modifications like sulfations, methylations, or acetylations (Bäumgen et al. 2021a). It was shown that complex enzymatic cascades are required for the breakdown of a single algal polysaccharide (Reisky et al. 2019; Sichert et al. 2020). Members of the bacterial phylum *Bacteroidetes* are considered specialists in the pivotal degradation of marine polysaccharides (Thomas et al. 2011a) and are observed as first responders after micro- and macroalgal blooms (Teeling et al. 2012; Brunet et al. 2021). They contain specific gene clusters referred to as polysaccharide utilization loci (PULs) (Grondin et al. 2017), which encode for carbohydrate-active enzymes (CAZymes) that catalyze the breakdown of the carbohydrates (Lapébie et al. 2019), as well as proteins essential for the binding and uptake of smaller sugar molecules (Bauer et al. 2006; Martens et al. 2009). Characterizing individual CAZymes helps elucidate the complete degradation pathways of marine carbohydrates and provides a deeper understanding of the global carbon cycle, which has been successfully performed, for instance, for ulvan from green algae (Reisky et al. 2019; Bäumgen et al. 2021b), fucoidan from brown algae (Sichert et al. 2020), and carrageenan from red algae (Ficko-Blean et al. 2017).

**Fig. 1:**
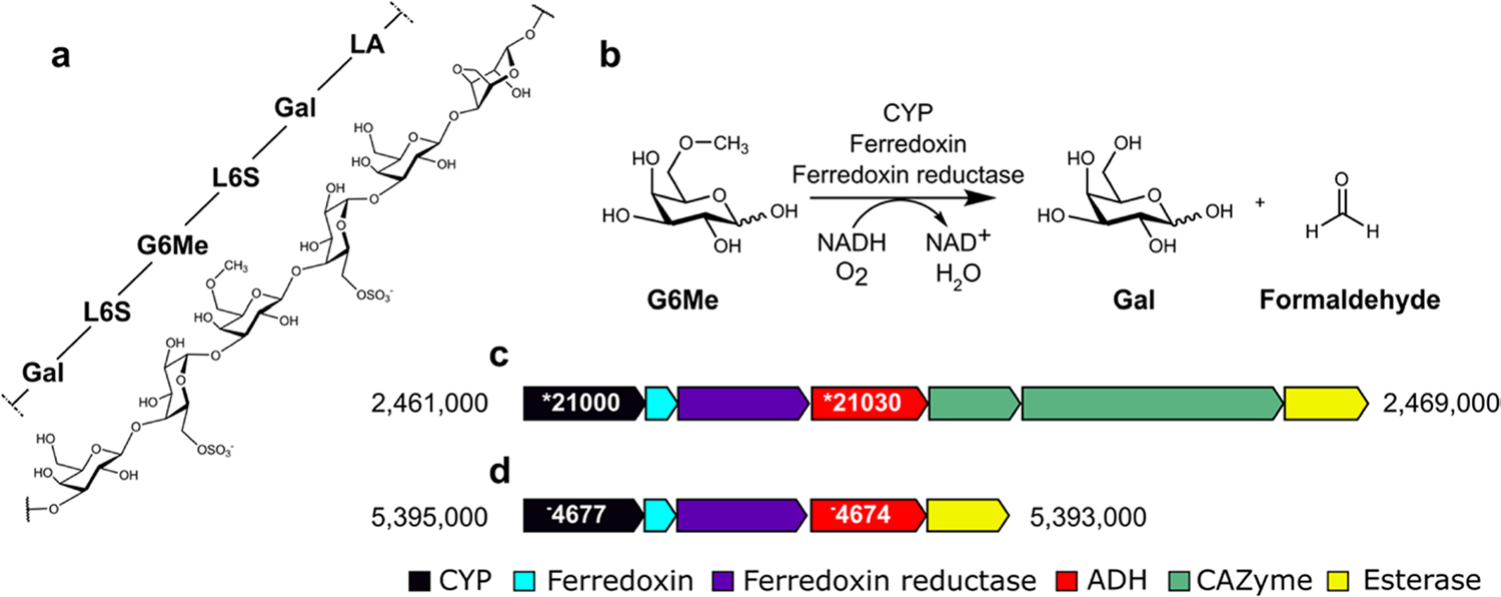
Porphyran contains 6-*O*-methyl-d-galactose, which can be metabolized by marine bacteria via oxidative demethylation. **a** Porphyran, the common name of the galactan of red algae of the genus *Porphyra*, consists of chains composed mainly of the alternating monosaccharide units 4-linked-α-l-galactose-6-sulfate (L6S) and 3-linked-β-d-galactose (Gal) or 3,6-anhydro-α-l-galactose (LA). Furthermore, the *O*-methylation of d-galactose results in the formation of 6-*O*-methyl-d-galactose (G6Me). **b** The oxidative demethylation of G6Me is catalyzed by a cytochrome P450 monooxygenase in combination with its redox partners ferredoxin and ferredoxin reductase, producing d-galactose and formaldehyde in equimolar amounts. **c** In *Formosa agariphila* KMM 3901^T^ and **d** *Zobellia galactanivorans* Dsij^T^, genes encoding for the key enzymes of oxidative demethylation are located in close proximity to a gene encoding for zinc-dependent alcohol dehydrogenase. *BN863_, for example, *21,030 refers to locus tag *BN863_21030* for *F. agariphila* while ^−^zgal, for example, ^−^4674 refers to locus tag *zgal_4674* for *Z. galactanivorans*

Recently, we have demonstrated that in the degradation process of the red algal galactan porphyran (Fig. 1a) by marine bacteria, oxidative demethylation of the methoxy sugar 6-*O*-methyl-d-galactose (G6Me) occurs (Reisky et al. 2018). This reaction, which is catalyzed by a cytochrome P450 monooxygenase (CYP) and its respective redox partners consisting of ferredoxin reductase and ferredoxin, leads to the formation of equimolar amounts of d-galactose and formaldehyde (Fig. 1b) (Reisky et al. 2018). It was hypothesized that this reaction is crucial in terms of G6Me utilization as it removes the highly stable methyl ether, consequently generating an easily metabolizable compound (Reisky et al. 2018). The crystal structure of the CYP from *Zobellia galactanivorans* Dsij^T^ provided additional information on the binding of G6Me as well as other mechanistic insights (Robb et al. 2018). In addition to the key enzymes for the oxidative demethylation of G6Me, glycoside hydrolases (GH2 and GH16), an esterase, and a putative zinc-dependent alcohol dehydrogenase (ADH) were also observed in the genomic context of the marine Flavobacterium *Formosa agariphila* KMM 3901^T^ (Fig. 1c) (Reisky et al. 2018). A similar genomic context was also found in *Zobellia galactanivorans* Dsij^T^ (Fig. 1d).

Considering the fact that dehydrogenases play only a minor auxiliary role in carbohydrate degradation and are poorly represented in the Carbohydrate-Active enZYmes (CAZy) database, with some exceptions in the AA3, AA6, AA7, and AA12 families (Takeda et al. 2015; Kracher and Ludwig 2016; Sützl et al. 2018), it remains unclear which biological function this ADH provides for the organism. ADHs belong to the enzyme class of oxidoreductases and catalyze the reversible oxidation of an alcohol to the corresponding aldehyde or ketone employing the nicotinamide adenine dinucleotide (NAD^+^) or nicotinamide adenine dinucleotide phosphate (NADP^+^) cofactor. Depending on the size of the substrate-binding domain, it is possible for ADHs to possess a broad substrate scope; while some exhibit only activities for small aliphatic alcohols, others can convert sterically challenging cyclic components (Persson et al. 2008; Sirota et al. 2021). A major family of ADHs includes the group of zinc-dependent ADHs, which exhibit a typical Rossmann fold (Rao and Rossmann 1973) and contain a catalytic zinc ion in the active site as well as additional non-catalytic zinc ion supporting the stability of an external loop structure (Hambidge et al. 2000). Various biological functions are observed within this family (Sirota et al. 2021), including polyol dehydrogenases catalyzing the conversion between sugar and sugar alcohol (Lu et al. 2019), cinnamyl alcohol dehydrogenases (Larroy et al. 2002; Pick et al. 2013), and glutathione-dependent formaldehyde dehydrogenases (Gutheil et al. 1992; Sanghani et al. 2000; Achkor et al. 2003), which play an important part in the detoxification of formaldehyde (Vorholt 2002). Additionally, ADHs provide numerous advantageous properties for organic synthesis, including high enantioselectivity and applicability under mild reaction conditions (Koesoema et al. 2020). Consequently, they are now employed in numerous biotechnological applications such as the preparation of chiral alcohols (Zhang et al. 2015), rare sugars (Lu et al. 2019), fine chemicals, as well as the synthesis of building blocks for various essential pharmaceuticals (Hall and Bommarius 2011; Zheng et al. 2017). Discovering and characterizing additional ADHs with unique biochemical properties is thus also desirable for potential industrial applications.

In this study, we aimed to elucidate the putative function of these ADHs, which are consistently located in close proximity to genes that are essential for the oxidative demethylation of G6Me of polysaccharide utilizing marine *Flavobacteriia*. We provide a detailed biochemical characterization as well as the crystal structures for the ADHs from *Formosa agariphila* KMM 3901^T^ (FoADH) and *Zobellia galactanivorans* Dsij^T^ (ZoADH). We propose the putative biological functions of these ADHs and demonstrate their importance for the utilization of G6Me via growth studies with a *Z. galactanivorans* knockout strain.

## Materials and methods

### Materials, strains, and plasmids

All chemicals and reagents used, unless otherwise specified, were purchased from Sigma-Aldrich (St. Louis, MO, USA), Thermo Fisher Scientific (Waltham, MA, USA), Th. Geyer (Berlin, Germany), ABCR GmbH (Karlsruhe, Germany), Honeywell Fluka™ (Morristown, NJ, USA), Carl Roth GmbH (Karlsruhe, Germany), chemPUR GmbH (Karlsruhe, Germany), TCI Deutschland GmbH (Eschborn, Germany), and Cayman Chemical Company (Ann Arbor, MI, USA). Porphyran and G6Me were obtained from Biosynth Carbosynth (Staad, Switzerland). Primers were obtained from Invitrogen (Waltham, MA, USA). Phage-resistant *Escherichia coli* (*E. coli*) BL21 (genotype: *fhuA2* [lon] *ompT gal* (λ *DE3*) [*dcm*] Δ*hsdS* λ *DE3* = λ *sBamHIo* Δ*Eco*RI-B int::(*lacI*::*PlacUV5*::*T7 gene1*) *i21* Δ*nin5*) was obtained from New England Biolabs (Ipswich, MA, USA). The conjugative strain *E. coli* S17-1 λ pir (genotype *λpir hsdR pro thi*; chromosomal integrated RP4-2 Tc::Mu Km::Tn7) (de Lorenzo and Timmis 1994) was grown from in-house glycerol stocks. A construct for the expression of the FoADH (GenBank accession number: OP548117) from *F. agariphila* KMM 3901^T^ was prepared using the FastCloning strategy (Li et al. 2011) with genomic DNA as a template for the amplification of the insert. *F. agariphila* KMM 3901^T^ (collection number DSM-15362) was obtained from the DSMZ (Braunschweig, Germany). The pET28a vector was amplified with the 5-GCG GCC GCA CTC GAG CA-3′ and 5-CAT ATG GCT GCC GCG C-3′ oligonucleotides, while the insert was amplified with the 5′-CAC AGC AGC GGC CTG GTG CCG CGC GGC AGC CAT ATG TCC ATA ATT TCA AAA TGC GCT ATT G-3′ and 5′-CAG TGG TGG TGG TGG TGG TGC TCG AGT GCG GCC GCT TAA AAA ATA ATT ACA CCC TTT GCA TTC-3′ oligonucleotides. A synthetic gene, codon optimized for expression in *E. coli*, encoding the ZoADH (GenBank accession number: OP548118) from *Z. galactanivorans* Dsij^T^, was synthesized and cloned into a pET28a vector by BioCat GmbH (Heidelberg, Germany). The constructs encode the recombinant protein as fusion to a N-terminal Strep-tag for affinity purification.

### Computational analysis for FoADH and ZoADH

Sequences of FoADH (Uniprot ID: T2KM87) and ZoADH (Uniprot ID: G0L712) were blasted against the MarDB and MarRef databases using the Marine Metagenomic Portal (Klemetsen et al. 2018; Priyam et al. 2019) with the − *e* value of 1e^−5^ and maximal target sequences of 1000. The automated fasta hit table of both blasts was fused and used for the generation of a sequence similarity network (Zallot et al. 2019). An alignment score of 150 was chosen for the refinement and generation of a genome neighborhood analysis of ten genes down- and upstream of the ADH genes (Zallot et al. 2019). Resulting diagrams were visualized via Cytoscape (Paul Shannon et al. 2003), and genome neighborhood diagrams were generated from the online server. Only shared sequences of the MarDB/MarRef database with the UniProtKB databases could be incorporated into the genome neighborhood analysis.

### ADH knockout in *Z. galactanivorans* and growth studies

The deletion mutant of the ADH gene *zgal_4674* in *Z. galactanivorans* Dsij^T^ (collection number DSM-12802) was constructed using a *sacB* system (Zhu et al. 2017), as previously described for the deletion variant of the CYP gene (Brott et al. 2022). Briefly, to delete *zgal_4674*, a 2448-bp fragment including the last 43 bp of *zgal_4674* and 2405 bp of the downstream sequence was amplified using primers OFT0041 and OFT0043 on genomic DNA from *Z. galactanivorans* Dsij^T^. The fragment was digested with *Bam*HI and *Xba*I and ligated into pYT313, which had been digested with the same enzymes, to generate pFT12. A 2077-bp fragment including the first 29 bp of *zgal_4674* and 2048 bp of the upstream sequence was amplified using primers OFT0040 and OFT0042. The fragment was cloned into *Xba*I and *Sal*I sites of pFT12 to generate the *zgal_4674* deletion construct pFT13. Conjugative transfer of pFT13 from *E. coli* S17-1 into the wild-type *Z. galactanivorans* Dsij^T^ and second recombination steps were carried out as described previously (Zhu et al. 2017). Deletions were confirmed by PCR and sequencing on isolated colonies using primer pairs OFT0044–OFT0045 to identify the *zgal_4674* deletion mutant (mZG_0080). Primers employed are displayed in Table S1 in the Supplementary Information (SI). For growth studies, precultures of three *Z. galactanivorans* strains (wild-type, knockout ADH, and knockout CYP) were prepared in the Zobell 2216E medium (Zobell 1941). The 3-day precultures were then rinsed twice with a sterile saline solution. Marine minimal medium (Thomas et al. 2011b) amended with d-galactose or G6Me (4 g L^−1^) was then inoculated so that an initial optical density (OD_600_) of 0.05 was achieved. Appropriate cultures were incubated for 3 days at room temperature.

### Enzyme production and purification

Chemically competent *E. coli* BL21 (DE3) cells were transformed with the plasmids harboring FoADH or ZoADH and were spread on lysogeny broth (LB) agar plates containing 50 µg mL^−1^ kanamycin. The agar plates were incubated overnight at 37 °C. One colony was picked and used to inoculate 5 mL LB medium which contained 50 µg mL^−1^ kanamycin and was then incubated at 37 °C and 180 rpm overnight. For overexpression, the cultivation was performed in a terrific broth (TB) medium containing 50 μg mL^−1^ kanamycin. The TB medium was inoculated with the overnight culture so that a starting OD_600_ of 0.05 was obtained. Cells were then incubated at 37 °C and 180 rpm until an OD_600_ of 0.8 was reached. Expression of target enzymes was induced by the addition of 1 mM isopropyl-β-d-thiogalactopyranoside (IPTG). The cultivation was performed at 25 °C and 180 rpm overnight. Cells were harvested by centrifugation at 10,000 × *g* and 4 °C for 1 h, washed with 50 mM sodium phosphate buffer (NaPi) pH 7.5, and subsequently stored at − 20 °C until cell disruption. The purification procedures of FoADH and ZoADH for crystallization and enzyme assays are identical. Cells were resuspended in 50 mM Tris–HCl buffer pH 8.0 containing 200 mM NaCl. Following cell lysis by ultra-sonication (2 × 3 min, 50% power, 50% cycle), cell debris was removed by centrifugation at 10,000 × *g*, at 4 °C for 20 min. The clarified supernatant was loaded on a gravity flow column containing Strep-Tactin XT Sepharose^®^ 50% suspension (IBA-Lifesciences GmbH, Göttingen, Germany) as column material. The column was washed with 100 mM Tris–HCl buffer pH 8.0 containing 150 mM NaCl in order to remove unbound and undesirable proteins. The target enzymes were then eluted with the same buffer containing an additional 50 mM of biotin. Elution fractions were pooled and concentrated using a Vivaspin 6 centrifugal concentrator with a 10 kDa molecular weight cut-off (Sartorius AG, Göttingen, Germany). Size exclusion chromatography was subsequently performed via the Äkta™ pure chromatography system (Cytiva Europe GmbH, Germany). The concentrated enzyme solution was applied to a HiPrep™ 16/60 Sephacryl^®^ S-200 HR column (Cytiva Europe GmbH, Freiburg, Germany) that was previously equilibrated with 10 mM Tris–HCl buffer pH 8.0 containing 200 mM NaCl. Elution fractions were collected, and the purity was verified by sodium dodecyl sulfate–polyacrylamide gel electrophoresis (SDS-PAGE). Pure fractions were combined and concentrated as mentioned above. The enzyme solution was stored at 4 °C for crystallization. For application in enzyme assays, a PD-10 desalting column (Cytiva Europe GmbH, Freiburg, Germany) was employed to desalt the protein sample and exchange the buffer.

### SDS-PAGE and determination of protein content

SDS-PAGE was performed to verify the purity of the target enzymes. Twenty microliters of protein sample was mixed with 5 µL of a fivefold stock of SDS sample buffer (100 mM Tris–HCl buffer at pH 6.8 containing 4% (w/v) SDS, 20% (v/v) glycerol, 2% (v/v) β-mercaptoethanol, 25 mM ethylenediaminetetraacetic acid (EDTA), and 0.04% (w/v) bromophenol blue) and denatured at 99 °C for 15 min. For the SDS-PAGE, a 12.5% acrylamide gel (separating gel) and a 4.0% loading gel were used. Electrophoresis was carried out at 200 V. Proteins were stained with Coomassie Blue (PhastGel^®^ Blue R). As a reference, the Pierce™ Unstained Protein Molecular Weight Marker (Thermo Fisher Scientific, Waltham, MA, USA) was used. Protein concentrations were determined using the Pierce™ BCA Protein Assay Kit (Thermo Fisher Scientific, Waltham, MA, USA) with bovine serum albumin as a protein standard.

### Crystallization

Purified FoADH (25 mg mL^−1^) and ZoADH (25 mg mL^−1^) were incubated with 20 mM NAD^+^ overnight. An initial crystallization screen was performed using the sitting drop vapor-diffusion method at 22 °C. The droplets contained 0.2 μL of protein and 0.2 μL of reservoir solution. Microcrystals of FoADH were obtained from a reservoir solution containing 0.1 M Tris–HCl at pH 7.5, 0.2 M KCl, and 22% (w/v) polyethylene glycol 3350. Microcrystals of ZoADH were obtained from a reservoir solution containing 0.1 M Tris–HCl at pH 7.5, 0.2 M KCl, and 20% (w/v) polyethylene glycol 3350. Further crystal optimization was performed by scale-up of the droplets containing 2 μL of protein and 2 μL of reservoir solution, using the hanging drop vapor-diffusion method at 22 °C. Suitable FoADH and ZoADH crystals for X-ray diffraction were obtained from 0.1 M Tris–HCl at pH 7.5, 0.2 M KCl, and 20–22% (w/v) polyethylene glycol 3350 within 1 day.

### Data collection

X-ray diffraction data were collected at beamline 11C at Pohang Light Source II (PLS-II, Pohang, South Korea) with a Pilatus 6 M detector (Dectris, Switzerland). The FoADH crystals were equilibrated in a cryoprotectant buffer containing reservoir buffer plus 20% (v/v) ethylene glycol. ZoADH crystals were equilibrated in a cryoprotectant buffer containing reservoir buffer plus 20% (v/v) glycerol. The crystal was mounted on the goniometer and cooled under a nitrogen gas stream at 100 K. The diffraction data were indexed, integrated, and scaled using the HKL2000 program (Otwinowski and Minor 1997). A data collection statistic is given in Table S2.

### Structure determination

The electron density maps of FoADH and ZoADH were obtained via the molecular replacement method using the MOLREP program (Vagin and Teplyakov 2010). The crystal structure of an ADH from *Artemisia annua* (PDB code: 6LJH, unpublished) was used as a search model for both FoADH and ZoADH. Model building and refinement were performed with the COOT program (Emsley and Cowtan 2004) and phenix.refinement in PHENIX (Liebschner et al. 2019), respectively. The geometry of the final models was evaluated with MolProbity (Williams et al. 2018). Structural figures were generated with PyMOL (www.pymol.org). Structure-based sequence alignments were generated using Clustal-Omega (Sievers et al. 2011) and ESPript (Gouet et al. 1999). Tetrameric interfaces of ADHs were analyzed by PDBePISA (Krissinel and Henrick 2007). The interaction between ADHs and ligands was analyzed using PLIP (Salentin et al. 2015). The structure factor and coordinates are deposited in the Protein Data Bank under PDB codes 8H2A (FoADH-NAD) and 8H2B (ZoADH-NAD).

### Enzyme activity determination and substrate screening

For determining the enzyme activity of the ADHs, the absorbance maximum of NADH at 340 nm was utilized. The absorbance at 340 nm was measured every minute over a 10-min period using a microplate spectrophotometer (BioTek Synergy H1, Agilent Technologies, Santa Clara, CA, USA), and the slope over time was used to determine activities or relative activities. One unit of activity is defined as the oxidation or formation of 1 µmol of NADH/min. For the calculation of activity, the molar absorption coefficient of NADH was determined via a standard curve that covered the range of 0 to 0.5 mM. For the initial substrate screening, several alcohols/aldehydes/ketones were employed at a final concentration of 10 mM. For increased substrate solubility, these reactions contained 3.5% (v/v) dimethyl sulfoxide (DMSO). The total volume for all reactions was 0.2 mL. The oxidation and reduction were both conducted at an incubation temperature of 70 °C. Reduction of aldehydes was performed in the presence of a 50 mM succinate buffer at pH 6.5, while oxidation reactions were assayed in the presence of a 50 mM NaPi buffer at pH 8.5. The final enzyme concentrations used to provide a linear absorbance increase or decrease ranged from 20 to 100 µg mL^−1^ for the oxidation reactions and from 0.25 to 2.5 µg mL^−1^ for the reduction reactions. The reaction was initialized by the addition of 0.5 mM NAD^+^ or NADH. For the measurement with sugar substrates, a reduced reaction temperature of 40 °C and an increased measuring time of 30 min were chosen. Various sugars were used at a final substrate concentration of 30 mM. A concentration of 0.2% (w/v) was used for porphyran. Oxidation and reduction reactions were performed in the identical buffers as used for substrate screening, and the final enzyme concentration was 0.1 mg mL^−1^. The reaction was initialized by the addition of 0.5 mM NAD^+^ or NADH. For the determination of cofactor utilization, the oxidation of 10 mM benzyl alcohol was performed in the presence of different NAD^+^ or NADP^+^ concentrations ranging from 0 to 5 mM in 50 mM HEPES buffer at pH 8.5 at 25 °C and a final enzyme concentration of 0.1 mg mL^−1^. For the determination of the kinetic parameters, a final protein content of 0.1 mg mL^−1^ (corresponding to a protein concentration of 2.44 µM) was used for the oxidation reactions. When determining *K*_m_ and *V*_max_ values for NAD^+^, 15 mM benzyl alcohol was used as the final substrate concentration, while a final cofactor concentration of 5 mM NAD^+^ was used for the measurement of benzyl alcohol. The oxidation reactions were carried out in 50 mM NaPi buffer at pH 8.5 and at a reaction temperature of 70 °C. A final protein content of 5 µg mL^−1^ was used in the reduction reaction (corresponding to a protein concentration of 0.012 µM). For the determination of the kinetic parameters for NADH, 2.5 mM pyridine-3-carbaldehyde was used as the final substrate concentration, while a final cofactor concentration of 0.5 mM NADH was used for the determination of the kinetic parameters for pyridine-3-carbaldehyde. The reduction reactions were carried out in 50 mM succinate buffer at pH 6.5 and at 70 °C. In order to test for thiol-dependent formaldehyde detoxification, different thiols were evaluated as potential cofactors. For this reaction, the thiol cofactor and formaldehyde were used in a 1:1 ratio at a final concentration of 0.5 mM. The measurement was performed in the 50 mM NaPi buffer at pH 8.5 at 70 °C with a final enzyme concentration of 0.2 mg mL^−1^. The reaction was started by the addition of 0.5 mM NAD^+^. The ADH-catalyzed disproportionation of formaldehyde into methanol and formate was monitored by a pH change utilizing the phenol red assay (Martínez-Martínez et al. 2018). This measurement was performed on a microtiter plate, and the reaction volume was 0.2 mL. Five millimolars of formaldehyde was used as a substrate, 0.5 mM NAD^+^ as a cosubstrate, and 0.1 mg mL^−1^ as the final enzyme concentration. The pH indicator phenol red was used at a final concentration of 91 µM. The reaction was performed in a 5 mM HEPES buffer at pH 8.5 at 40 °C. Absorbance at 560 nm was measured every minute for 20 min.

### Influence of pH and buffer components

To determine the pH optimum of the enzymes, the oxidation and reduction reactions were both investigated in the presence of varying pH values. All buffers had a concentration of 50 mM. A citrate buffer was used in the pH range of 5 to 6, a NaPi buffer in the range of 6 to 8.5, a CHES buffer in the range of 8.5 to 10, and a CAPS buffer in the range of 10 to 12.5. The assay conditions for the oxidation reaction were as follows: 200 µL reaction volume, 10 mM benzyl alcohol, and 0.5 mM NAD^+^ was used as substrate. The reaction was started by the addition of 0.1 mg mL^−1^ ADH. For the reduction reaction, instead of benzyl alcohol and NAD^+^, 10 mM benzaldehyde and 0.5 mM NADH were used. Since benzaldehyde was less soluble in the buffer than benzyl alcohol, both reactions contained 3.5% (v/v) DMSO in order to achieve better comparability. The reaction was carried out at 25 °C in the respective buffers. To examine the influence of buffer components on enzyme activity, different buffers with a concentration of 50 mM were used. The buffers had a pH of 6.5 for the reduction reaction, whereas it was 8.5 for the oxidation reaction. The reaction was carried out under the same conditions as those for the pH optimum. Relative activities were determined as described above.

### Influence of temperature and thermostability

The temperature optimum was determined by conducting the oxidation reaction at different temperatures in the range between 20 and 90 °C. For this, the reaction mixture without enzyme was preheated to the desired temperature in a reaction tube by using a heating block (Eppendorf ThermoMixer^®^C, Eppendorf SE, Hamburg, Germany) for at least 45 min. The reaction mixture had a volume of 200 µL. Thirty millimolars of benzyl alcohol and 0.5 mM of NAD^+^ were employed as substrates, and the reaction was carried out at different temperatures ranging from 20 to 90 °C in a 50 mM NaPi buffer at pH 7.5. The reaction was initiated by the addition of an enzyme with a final concentration of 0.1 mg mL^−1^. For the thermostability determination, the purified ADH (1 mg mL^−1^) was incubated in 50 mM NaPi buffer at pH 7.5 for 1 or 4 h in a gradient thermal cycler (FlexCycler^2^, Analytik Jena, Jena, Germany) at various temperatures ranging from 20 to 80 °C. Residual activity was then determined as described above and compared with a control that was incubated on ice. The assay conditions were as follows: the reaction volume was 200 µL, the final enzyme concentration was 0.1 mg mL^−1^, the substrate was 10 mM benzyl alcohol, and the reaction was performed at 40 °C in 50 mM NaPi buffer at pH 7.5. The reaction was initiated by the addition of 0.5 mM NAD^+^.

### Influence of sodium chloride

The determination of NaCl influence on enzyme activity was performed by carrying out the oxidation reaction in the presence of different NaCl concentrations varying from 0 to 800 mM. The relative activities were determined as described above and were compared with the control, where no additional NaCl was present. Assay conditions were as follows: the reaction volume was 200 µL, the substrate was 10 mM benzyl alcohol, the final enzyme concentration was 0.1 mg mL^−1^, and the NaCl concentration was between 0 and 800 mM. The reaction was carried out at 25 °C in a 50 mM NaPi buffer at pH 8.5 or in a 50 mM tricine buffer at pH 8.5 and started by the addition of 0.5 mM NAD^+^.

### Influence of metal ions and other small molecules

For the determination of the influence of various metal ions on enzyme activity, the ADHs with a concentration of 1 mg mL^−1^ were incubated with either 1 or 10 mM metal ions at RT for 1 h before activity measurement. A sample without additional metal ions served as a control. For the activity measurement, the standard assay was used under the following conditions: the reaction mixture had a total volume of 200 µL, a substrate of 10 mM benzyl alcohol was used, a final enzyme concentration of 0.1 mg mL^−1^ was employed, and the reaction was performed in 50 mM HEPES buffer at pH 8.5 at 25 °C. The reaction was initiated by the addition of 0.5 mM NAD^+^. In order to determine the effect of EDTA, dithiothreitol (DTT), and 2-mercaptoethanol (2-ME) on enzyme activity, the ADHs were incubated at a protein concentration of 1 mg mL^−1^ with these components at concentrations of 1, 10, or 25 mM for 1 h at RT before activity determination. Higher concentrations of up to 100 mM were additionally tested for EDTA. The untreated enzyme served as a control. The activity measurement was performed as described for the influence of metal ions.

### Influence of solvents and formaldehyde

To evaluate the influence of selected water-miscible solvents on the activity of both ADHs, the oxidation reaction was conducted in the presence of 5, 10, and 20% (v/v) solvent and compared with a control containing no additional solvent. The relative activity was determined as described above. The total reaction volume was 0.2 mL, and 0.1 mg mL^−1^ of the enzyme was used as the final enzyme concentration. The reactions were performed in 50 mM NaPi buffer at 25 °C. Ten millimolars of benzyl alcohol was employed as a substrate, and the reactions were started by adding 0.5 mM NAD^+^. The enzymes were incubated at a concentration of 1 mg mL^−1^ with different concentrations of formaldehyde varying from 0 to 50 mM for 1 h at RT prior to activity measurement to evaluate the effect of formaldehyde on enzyme activity. Relative activity was determined as described above. For the activity measurement, the same conditions were used for the influence of solvent.

## Results

### Distribution and gene neighborhood analysis

In order to obtain an overview regarding the distribution and function of these ADHs in marine bacteria, we queried the MarDB and MarRef databases for ADHs with similar sequences to FoADH and ZoADH and constructed a sequence similarity network based on an alignment score of 150 and a sequence identity of 63.14%. This analysis revealed six main clusters, which we define here as clusters containing at least 34 sequences, with FoADH and ZoADH included in main cluster 2 (Fig. S1). This main cluster primarily contained sequences that were annotated as zinc-dependent ADHs, histidine kinases, ADH GroES-like domains, and some glutathione-dependent formaldehyde dehydrogenases/ADHs. However, glutathione-dependent and mycothiol-dependent formaldehyde dehydrogenases were identified predominantly in clusters 1 and 4, respectively. Based on main cluster 2, we performed a genome neighborhood analysis to obtain a general sense of which genes are located in close proximity to the ADH gene. Similar genomic arrangements consisting of CYP, redox partners, an esterase, and the ADH can be identified in several marine bacteria that are capable of degrading marine polysaccharides (Fig. S2), including members of the genera *Polaribacter*, *Maribacter*, and *Arenibacter*. Minor differences in gene arrangement can be observed among some organisms such as *F. agariphila* or *Algibacter lectus*, where genes encoding for CAZymes (GH2 and GH16) are located between the ADH and the esterase gene. Additionally, some genes encoding for sulfatases and SusC/SusD homologs, which are responsible for the binding and transport of sugar molecules (Martens et al. 2009), are located up- and downstream of the ADH gene. Considering that the ADH gene consistently appears in the proximity of the genes, which encode for CAZymes and key enzymes for the oxidative demethylation of G6Me, it is conceivable that the ADH possesses a specific function in carbohydrate utilization or a subsequent reaction.

### Knockout of the ADH encoding gene in *Z. galactanivorans* and growth studies

**Fig. 2:**
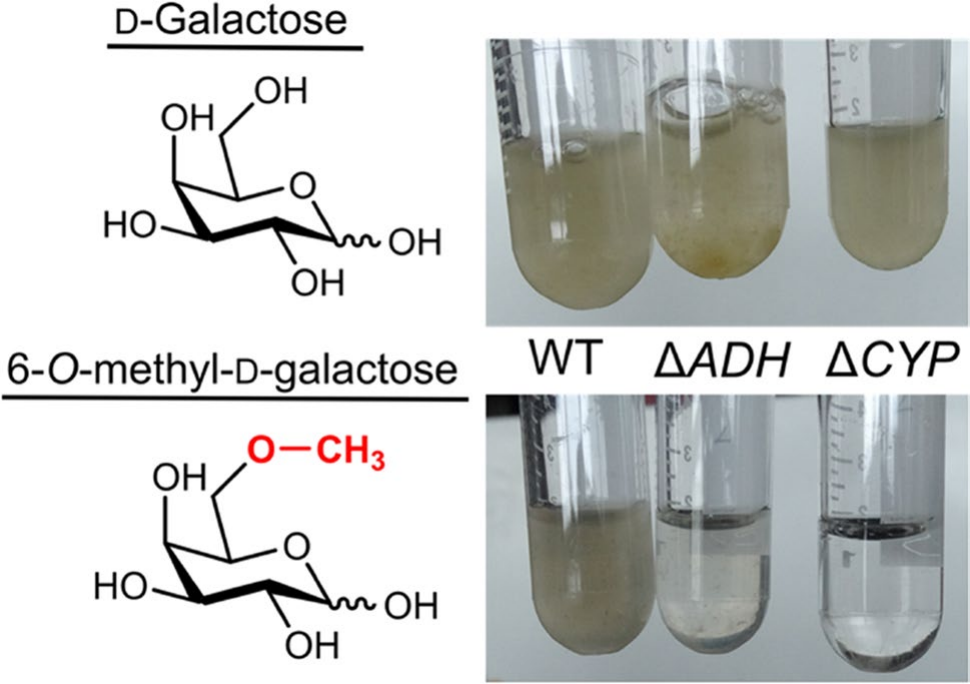
Knockout of the ADH gene in *Z. galactanivorans* leads to impaired growth on G6Me. Different *Z. galactanivorans* strains (wild type (WT), gene knockout ADH (Δ*ADH*), and gene knockout CYP (Δ*CYP*)) were incubated in minimal medium amended with d-galactose or G6Me for 3 days at RT

In an attempt to elucidate the biological relevance of the ADHs for the organisms, a knockout of the gene that encodes for the ADH in *Z. galactanivorans* was performed, followed by growth experiments. The controls employed for these growth studies were the wild-type (WT) and an additional knockout strain of *Z. galactanivorans* in which the CYP gene was deleted. When G6Me was employed as the sole carbon source, impaired growth was observed for the ADH and CYP knockout strains, while the WT exhibited normal growth (Fig. 2). In contrast, regular growth was observable for all three strains in a control, which contained d-galactose as sole carbon source. Consequently, the ADH possessed an impact on the G6Me utilization of *Z. galactanivorans*.

### Functional overexpression and purification of the ADHs

Since we could demonstrate the biological significance of the ADH for the utilization of G6Me by the gene knockout in *Z. galactanivorans*, our next aim was to identify the enzyme function. We, therefore, cloned the gene encoding for the ADH from *F. agariphila* into a pET28a vector. For the ADH from *Z. galactanivorans*, a synthetic gene was ordered in the pET28a vector. Both enzymes were successfully overexpressed and purified (Fig. S3), which established the basis to elucidate the putative biological functions of these ADHs by performing biochemical and structural biological characterizations.

### Substrate spectrum of the ADHs

In order to obtain a preliminary understanding of the substrate spectrum of these ADHs, their ability for alcohol oxidation as well as the reduction of various aldehydes and ketones were examined. Both enzymes converted predominantly aromatic substrates (Tables 1 and 2). The highest specific activity of 64.1 U mg^−1^ for FoADH and 54.9 U mg^−1^ for ZoADH was observed for the reduction of pyridine-3-carbaldehyde. In addition to compounds containing a benzene ring, substrates harboring a furan or thiophene ring, such as furfural and thiophene-3-carbaldehyde, were also preferentially converted. Positions of additional substituents at the benzene ring influenced the activity. A difference in the specific activities for the constitutional isomers of terephthalaldehyde and tolualdehyde was observed for both enzymes. In particular, substrates that possessed an additional substituent in *ortho*-position were converted significantly less efficiently. In addition, the length of the aldehyde substituent at the benzene ring also affected the activity. For instance, hydrocinnamaldehyde was converted by both enzymes, whereas for phenylacetaldehyde, no activity was observable. In contrast to benzaldehyde, the structurally similar acetophenone could not be oxidized. Thus, both ADHs were unable to convert ketones to secondary alcohols. In comparison to the reduction reaction, significantly reduced specific activities were noticed for the oxidation reactions (Table 2). Simultaneously, lower *K*_m_ values in the range of 0.6 to 0.8 mM could be determined for pyridine-3-carbaldehyde compared to the *K*_m_ values of 3.6 and 5.3 mM for benzyl alcohol (Fig. S4). The highest specific activity of 490 mU mg^−1^ for FoADH and 290 mU mg^−1^ for ZoADH has been observed for 2,5-bis(hydroxymethyl)furan. Both ADHs lacked any activity for smaller aliphatic alcohols such as methanol and ethanol. Since the ADHs exhibited predominantly activities for substrates containing a ring structure, several sugars were also considered possible substrates. However, no activity was observed for the oxidation or reduction of galactose, G6Me, and additional monosaccharides and disaccharides (Table S3). Additionally, the marine carbohydrate porphyran was also evaluated as a potential substrate; however, no activity was detected either. As mentioned earlier in the “Introduction,” ADHs require either NAD^+^ or NADP^+^ as a cofactor for their enzymatic activity. In order to identify the preferred cofactor for both ADHs, the oxidation of benzyl alcohol was conducted in the presence of varying NAD^+^ and NADP^+^ concentrations. Both ADHs utilize NAD^+^ as a cofactor, whereas in the presence of up to 5 mM NADP^+^, no activity for the oxidation reaction was observed.

**Table 1:**
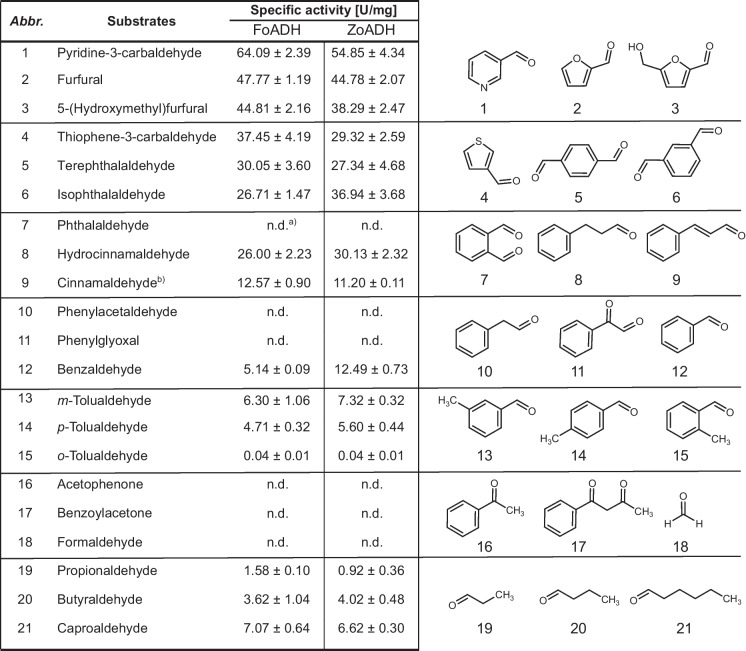
Initial substrate screening of the ADH in the reduction direction revealed that it preferentially converts aromatic aldehydes

Substrates were employed at a final concentration of 10 mM. For NADH, a concentration of 0.5 mM was used. The reaction contained 3.5% (v/v) DMSO. The reaction was conducted in a 50 mM succinate buffer at pH 6.5 at an incubation temperature of 70 °C. All measurements were performed in triplicates; the mean and the standard deviation are given

*n.d.*, not detected

^a^Due to a high background absorption of the compound, a substrate concentration of 1 mM was employed

**Table 2:**
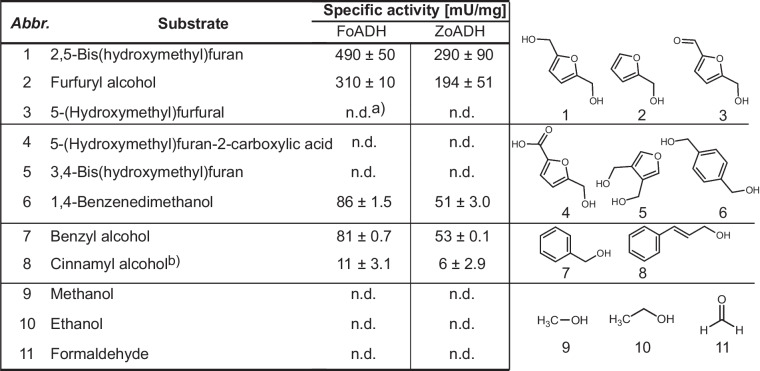
Both ADHs possess minor, specific activities for the oxidation of alcohols

Formaldehyde was also tested in a possible oxidation reaction to exclude thiol-independent formaldehyde dehydrogenase activity. Substrates were employed at a final concentration of 10 mM. For NAD^+^, a concentration of 0.5 mM was used. The reaction contained 3.5% (v/v) DMSO. The reaction was conducted in a 50 mM NaPi buffer at pH 8.5 at an incubation temperature of 70 °C. All measurements were performed in triplicates; the mean and the standard deviation are given *n.d.*, not detected

^a^Due to a high background absorption of the compound, a substrate concentration of 1 mM was employed

### Testing for formaldehyde detoxification activity

**Fig. 3:**
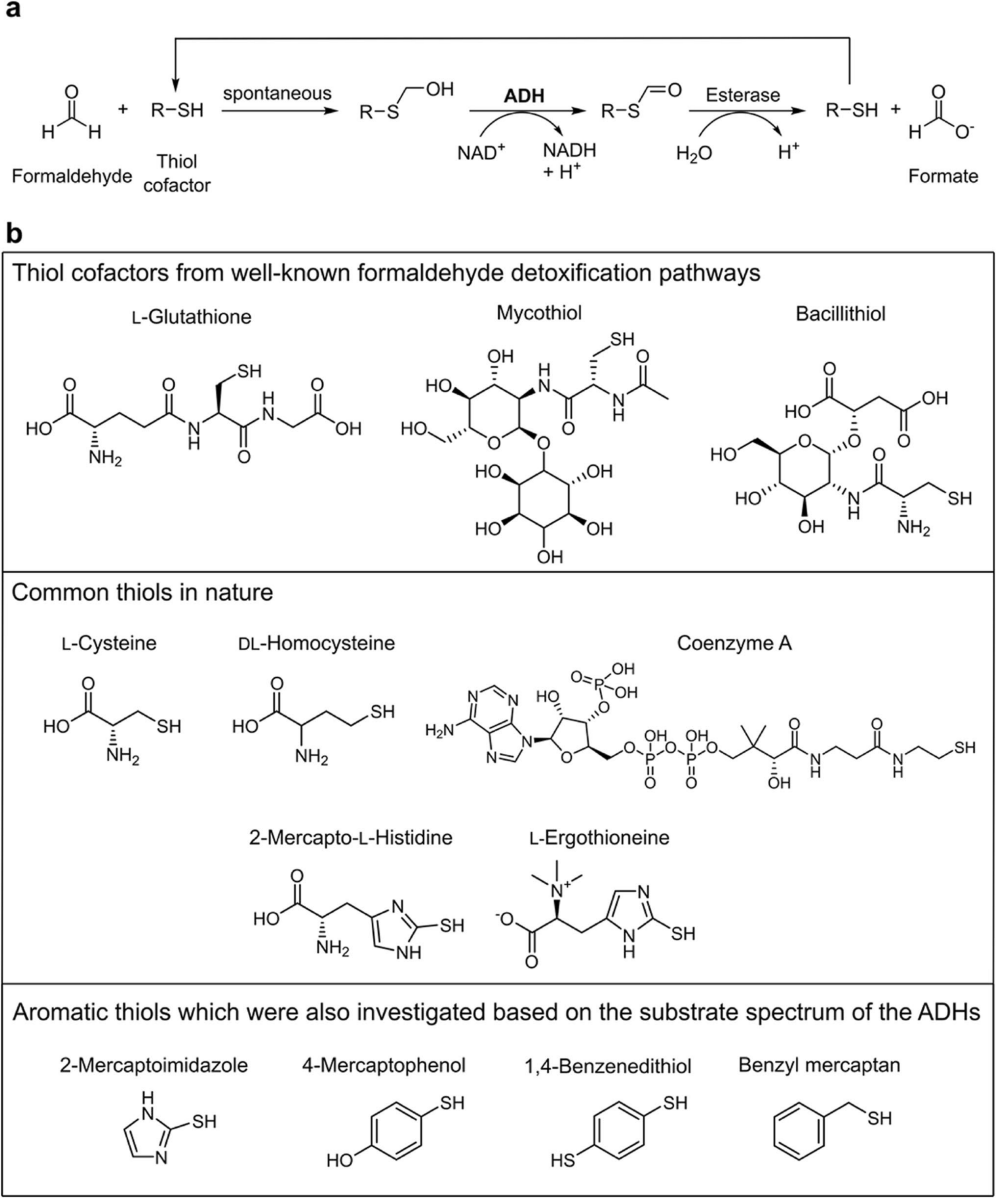
Thiol-dependent detoxification of formaldehyde catalyzed by an ADH and an esterase. **a** Principle of thiol-dependent detoxification of formaldehyde and **b** investigated thiols

Since the activity was neither observed for galactose nor for G6Me, we hypothesized that the ADHs may participate in formaldehyde detoxification, considering that formaldehyde is formed as a by-product in the oxidative demethylation reaction. Members of the zinc-dependent ADHs may catalyze the glutathione-dependent formaldehyde detoxification; therefore, various thiols were considered potential cofactors. Thiol-dependent detoxification of formaldehyde proceeds via a spontaneous reaction between the sulfhydryl group of the thiol cofactor and the carbon atom of formaldehyde, resulting in the formation of an alcohol (Fig. 3a) (Chen et al. 2016). Subsequently, this alcohol can be oxidized by the ADH to a thioester, which is then converted by an esterase to formate and the starting thiol cofactor (Gonzalez et al. 2006). Based on the results of our genome neighborhood analysis, where we have also demonstrated that a gene encoding for an esterase is located in the vicinity of the ADH gene, it is quite possible that thiol-dependent detoxification of formaldehyde can proceed via both enzymes. In addition to glutathione, mainly mycothiol (Misset-Smits et al. 1997; Newton and Fahey 2002) and bacillithiol (Newton et al. 2009; Chandrangsu et al. 2018) are well-known cofactors in formaldehyde detoxification (Fig. 3b). However, no activity was detected for these thiols. Furthermore, common thiols abundant in nature such as cysteine, coenzyme A, and l-ergothioneine (Hand and Honek 2005) were also investigated as cofactors. Nevertheless, no activity was observed for these substrates in combination with formaldehyde either. Considering that the ADHs mainly exhibited activity for aromatic substrates, aromatic thiols such as 2-mercaptoimidazole or 4-mercaptophenol were considered possible substrates as well. However, even with these compounds, no oxidation reaction was detected. Furthermore, neither enzyme exhibited activity for the oxidation or reduction of formaldehyde in the presence of only NAD^+^ or NADH as cofactors. In addition, a disproportionation reaction of formaldehyde into methanol and formate catalyzed by the ADH was also checked. However, no activity could be detected. Consequently, the ADHs possessed no activities for the substrate or for the products of the oxidative demethylation of G6Me. To provide additional insights into these ADHs, we performed further biochemical characterizations of both enzymes.

### Influence of pH and buffer components on enzyme activity

**Fig. 4:**
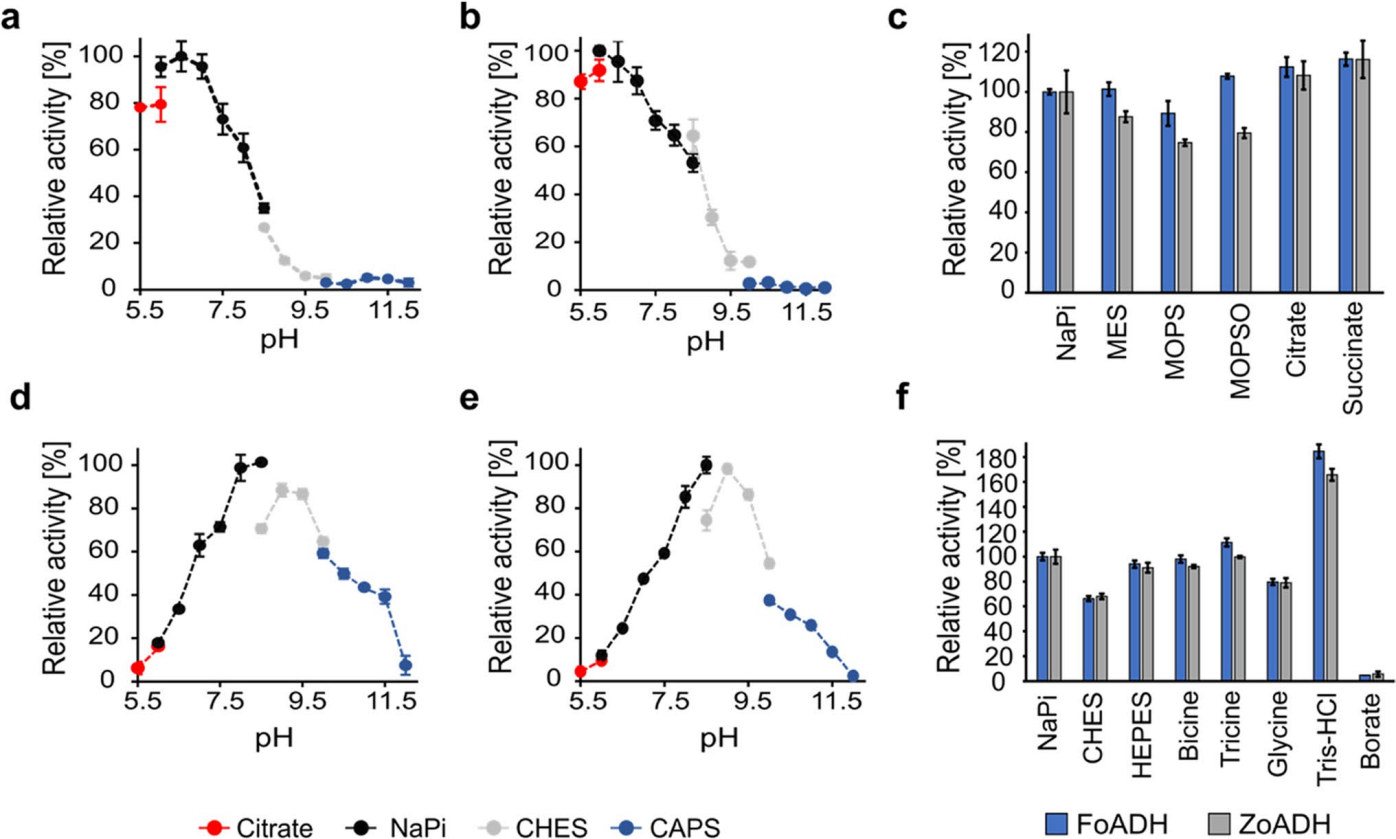
Influence of pH and buffer components on the ADH activity. pH optimum for the reduction reaction of **a** FoADH and **b** ZoADH as well as the pH optimum for the oxidation reaction catalyzed by **d** FoADH and **e** ZoADH. **c** Reduction of benzaldehyde and **f** oxidation of benzyl alcohol by the ADHs at the respective pH optima using various buffers. A pH of 6.5 was employed for the reduction reaction and a pH of 8.5 for the oxidation reaction; all buffers had a concentration of 50 mM. Since some buffers including bicine, tricine, Tris, MOPSO, and HEPES contain hydroxyl groups, a falsified activity due to the turnover of these substances was excluded by a measurement without additional substrate. However, no activity was observed for any buffer component. All measurements (**a**-**f**) were performed under the following conditions: a final substrate concentration of 10 mM benzyl alcohol or benzaldehyde, 3.5% (v/v) DMSO, and 0.5 mM NAD^+^ or NADH was used. The reaction was started by the addition of ADH at a final enzyme concentration of 0.1 mg mL^−1^. The measurement was performed at 25 °C in the respective buffers with concentrations of 50 mM. The maximum relative activity (100%) corresponds to the measurements in the 50 mM NaPi buffers at pH 6.5 for reduction and pH 8.5 for oxidation reactions. All measurements were performed in triplicates; the mean is given, and the error bars indicate the standard deviation

In order to determine the optimal pH for the enzymatic reaction, several buffers were investigated in the pH range from 5.5 to 12.5. A similar pH optimum was observed for both enzymes (Fig. 4). The reduction reaction was most efficiently catalyzed at pH 6.5, while oxidation was found to be most efficient at pH 8.5 (Fig. 4a, b, d–e). At pH 5 and at 12.5, no activity was detected for either enzyme; precipitation was noticed at pH 5 while employing higher protein concentrations. Since a considerable difference in activity was observed between NaPi and CHES buffer at pH 8.5, other buffers were also evaluated at pH 6.5 (Fig. 4c) and 8.5 (Fig. 4f) to investigate the influence of buffer components on the activity. For the oxidation reaction at pH 8.5, it was shown that by employing a Tris–HCl buffer, an approximately 60 to 80% increased activity was obtained compared to the activity in the NaPi buffer. In contrast, a significant activity decrease of 95% was observed for both enzymes in the presence of a borate-NaOH buffer. For the reduction reaction at pH 6.5, a slight increase in activity of ~ 8 to 16% could be detected using citrate and succinate buffer compared to the NaPi buffer, with the highest activity found for the succinate buffer.

### Influence of temperature and enzyme thermostability

**Fig. 5:**
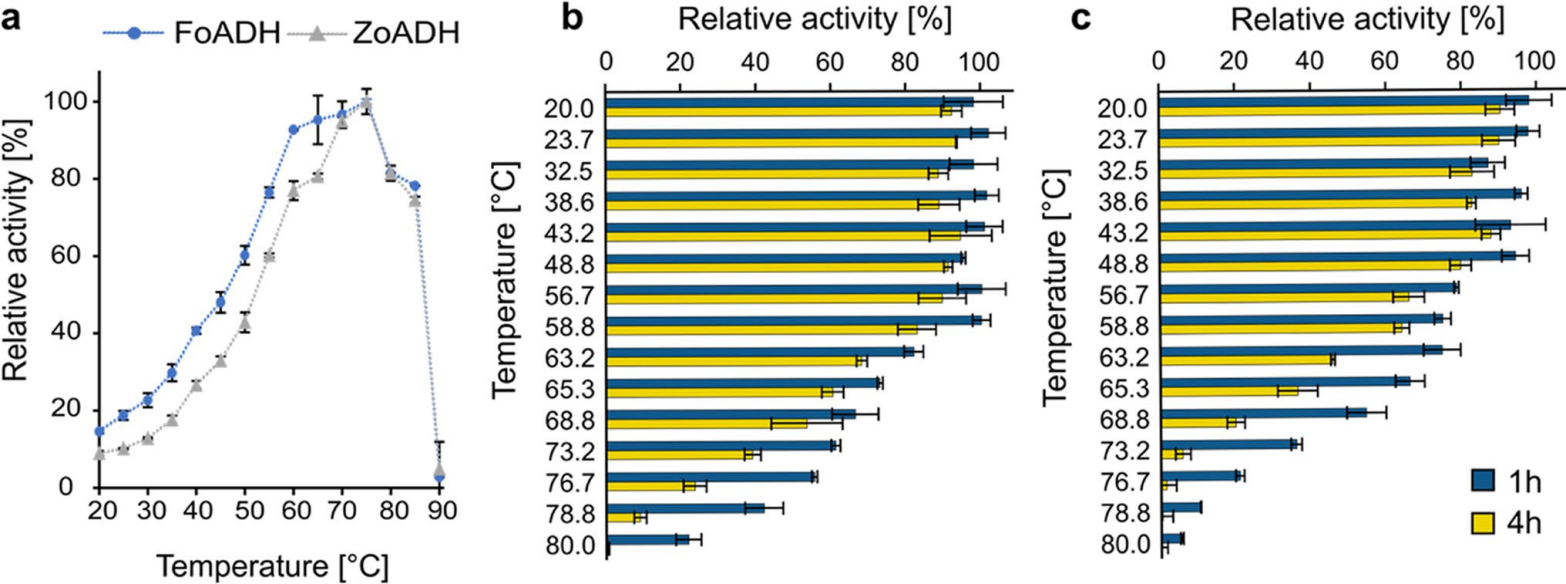
Temperature optimum and thermostability of the ADHs. **a** Influence of temperature on enzyme activity. The measurement was performed at various temperatures ranging from 20 to 80 °C for 10 min. The maximum relative activity (100%) corresponds to the measurement at 75 °C for both enzymes. Influence of temperature on enzyme stability for **b** FoADH and **c** ZoADH. The enzymes with a concentration of 1 mg mL^−1^ were incubated at different temperatures between 20 and 80 °C for 1 or 4 h, followed by the determination of residual activity. The measurement was performed at 40 °C. The maximum relative activity (100%) corresponds to a control incubated on ice for 1 or 4 h. All measurements (**a**-**c**) were performed under the following conditions: a final substrate concentration of 10 mM benzyl alcohol and 0.5 mM NAD^+^ was used. The reaction was started by the addition of ADH at a final enzyme concentration of 0.1 mg mL^−1^. The measurements were performed in a 50 mM NaPi buffer at pH 7.5. All measurements were performed in triplicates; the mean is given, and the error bars indicate the standard deviation

In addition to the pH value, the temperature influence is essential for enzymatic activity. At the same time, elevated temperatures promote substrate solubility and thus the application of higher concentrations, which may also shift the reaction equilibrium towards product formation (Unsworth et al. 2007). Therefore, the impact of temperature in the range between 20 and 90 °C was investigated for both enzymes. The ADHs possessed a similar temperature profile, where activity increased with rising temperature, reaching an optimum between 65 to 75 °C (Fig. 5a). However, at higher temperatures, the activity decreased rapidly, whereas at room temperature, only a relative activity of about 18% for FoADH and 10% for ZoADH was observed. The measurement for the temperature optimum was performed for 10 min to ensure that any influence of thermostability would not affect the results. The thermostability of enzymes is an important parameter for biocatalysis since many industrial processes operate at higher temperatures for longer time periods, leading to increased product yields. The thermostability of the ADHs was therefore evaluated next by incubating the enzymes for 1 or 4 h at various temperatures ranging from 20 to 80 °C followed by determination of residual activity. After 1 h incubation at 59 °C as well as lower temperatures, no decrease in activity was detected for FoADH compared to a control incubated on ice (Fig. 5b). Residual activity only diminished at higher incubation temperatures, and residual activity of roughly 20% was still observed for 80 °C. In contrast, after 4 h incubation, almost no residual activity was observed at this temperature. Nevertheless, even after this extended incubation period, a high remaining activity of approximately ≤ 85% was detected for the temperature range of 20 to 59 °C. ZoADH exhibited a similar behavior in thermostability as FoADH; however, an initial activity decrease of 20% was observed for the 1 h incubation already at 57 °C (Fig. 5c). A severe activity loss of almost 95 to 100% was observed for ZoADH when incubated for 4 h at temperatures ˃73 °C.

### Influence of sodium chloride

Enzymes originating from marine organisms may possess habitat-related characteristics such as an increased salt tolerance (Trincone 2011). Considering that both enzymes originate from marine bacteria, the influence of NaCl on the enzyme activity was tested. For this purpose, the relative activities for the oxidation reaction were determined in the presence of different NaCl concentrations ranging from 0 to 800 mM in the NaPi and tricine buffer, respectively. Both ADHs displayed similar behavior in the presence of rising NaCl concentrations (Fig. S5). An increase in the relative activity of approximately 10% was observed in the range from 0 to 150 mM NaCl for FoADH using the tricine buffer. In contrast, only a minor increase in activity was observed for the NaCl concentration of 100 mM in the NaPi buffer. A difference in the NaCl influence depending on the selected buffer was also noticed for ZoADH, with a higher effect in the tricine buffer. For ZoADH, an increase in the relative activity of 20% was also detected in the range of 0 to 200 mM NaCl. At NaCl concentrations ≥ 400 mM, a diminished relative activity was observed for both enzymes.

### Influence of metal ions and other small molecules

Both enzymes are annotated as zinc-dependent ADHs, which contain a catalytic zinc ion in the active site. An influence of various metal ions on the enzyme activity is thus possible and was therefore investigated next. For this purpose, the enzymes were incubated with different metal ions at concentrations of 1 or 10 mM for 1 h prior to activity measurement, and the relative activities were determined. High dependence on metal ions was observed for both ADHs, with nearly all ions assayed exhibiting a beneficial effect on enzyme activity (Table 3; Fig. S6). Particularly higher concentrations of Ni^2+^, Co^2+^, and Mn^2+^ led to a 10- to 14-fold increase in relative activity for both enzymes compared to the control, which contained no additional metal ion. In contrast, complete inhibition for both enzymes was only observed for Cu^2+^, Zn^2+^, and 10 mM Fe^3+^. Additionally, we analyzed whether the chelating agent EDTA, which is capable of complexing bivalent metal ions, affects the enzymatic activity. After 1 h incubation in the presence of 25 mM EDTA, a reduction in the relative activity for both enzymes was found, while an almost complete inhibition was observable at an EDTA concentration of 100 mM (Table 3; Fig. S7). The influence of DTT and 2-ME on activity was also investigated since these compounds can affect enzyme stability. DTT had a lesser impact on both enzymes than 2-ME. A major decline in the relative activity of over 70% was observed for both enzymes after 1 h incubation with 10 mM 2-ME (Table 3; Fig. S7). When compared to ZoADH, the effect of the reducing agents was more pronounced for the activity of FoADH.

**Table 3:** Influence of various substances on the enzyme activity of both ADHs

Chemical	Conc. (mM)	Relative activity (%)	
		FoADH	ZoADH
None	–	100 ± 1.3	100 ± 9.3
KCl	10	121 ± 1.9	154 ± 7.9
CaCl_2_	10	188 ± 6.3	306 ± 9.9
MgCl_2_	10	219 ± 7.2	328 ± 11.7
NiCl_2_	10	891 ± 13.8	1004 ± 6.6
CoCl_2_	10	1280 ± 38.1	1242 ± 25.4
MnCl_2_	10	1394 ± 58.5	973 ± 45.0
ZnCl_2_	10	n.d	n.d
CuCl_2_	10	n.d	n.d
FeCl_3_	10	n.d	n.d
EDTA	25	61 ± 1.9	61 ± 7.8
DTT	10	48 ± 4.6	85 ± 3.9
2-ME	10	19 ± 1.7	28 ± 4.1

The ADH was incubated with the respective component for 1 h at RT prior to measurement. The maximum relative activity (100%) corresponds to the measurement for the control, which contained no additives. All measurements were performed under following conditions: a final substrate concentration of 10 mM benzyl alcohol and 0.5 mM NAD^+^ was used. The reaction was started by the addition of ADH at a final enzyme concentration of 0.1 mg mL^−1^. The measurements were performed in a 50 mM HEPES buffer at pH 8.5 at 25 °C. All measurements were performed in triplicates; the mean and the standard deviation are given

*n.d.*, not detected

### Influence of solvents and formaldehyde

The influence of water-miscible solvents on the enzyme activity of both ADHs was also investigated. Increasing the amount of solvent in the reaction led to a decrease in the relative activity for all tested solvents (Fig. S8). Compared to the other solvents, methanol and DMSO had the weakest negative effects on the enzyme activity, leading to a relative activity of still 50% in the presence of 10% (v/v) solvent. In addition, the presence of formaldehyde on the enzyme activity was examined, since formaldehyde is released during the oxidative demethylation of G6Me and the ADHs are most likely involved in this reaction. Therefore, the ADHs were incubated with a variety of formaldehyde concentrations in the range between 0 and 50 mM for 1 h at RT, and the relative activities were determined. In the presence of 0 to 1 mM formaldehyde, no reduction in activity was observed. An initial decrease in the relative activity of approximately 10–20% could be perceived in the presence of 2.5 mM formaldehyde (Fig. S9). At higher formaldehyde concentrations, a more severe activity decrease was found, while no activity was observed for both enzymes in the presence of 50 mM formaldehyde.

### Overall structures of FoADH and ZoADH

**Fig. 6:**
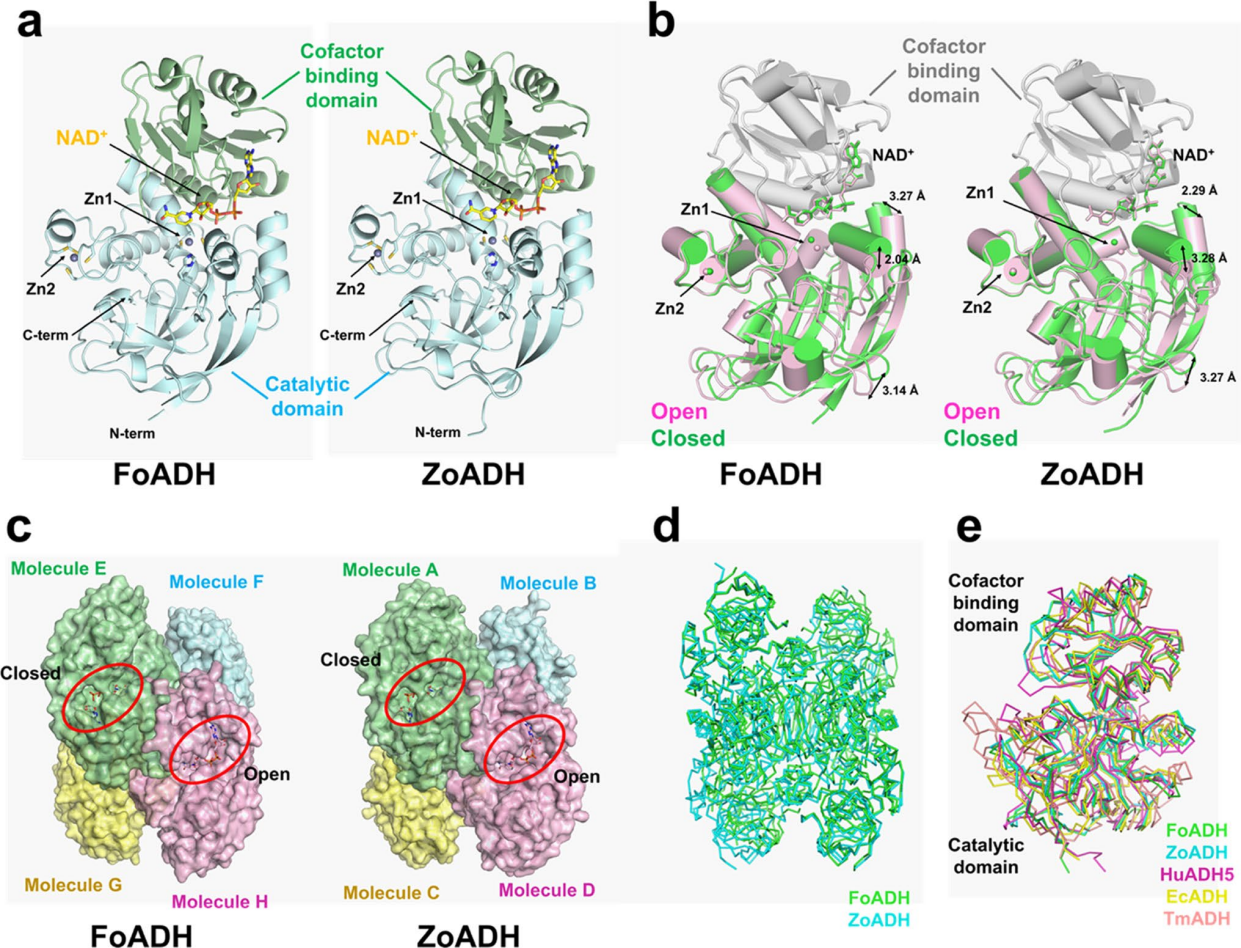
Crystal structures of FoADH and ZoADH. **a** Monomer structures of ZoADH and FoADH. The catalytic and cofactor domains are indicated by cyan and green, respectively. NAD^+^ and zinc ions are indicated by a yellow stick and a gray sphere, respectively. **b** Superimposition of closed (green) and open conformation between catalytic and cofactor-binding domains of ZoADH and FoADH monomers. The superimposed cofactor-binding domain of ZoADH and FoADH are indicated as gray cartoons. **c** Tetrameric formation of ZoADH and FoADH. **d** Superimposition of tetrameric formation of FoADH (green) and ZoADH (cyan). **e** Superimposition of monomer structures of FoADH (green) and ZoADH (cyan) with all-trans-retinol dehydrogenase ADH4 from *Homo sapiens* (pink, PDB code: 3COS), uncharacterized zinc-type alcohol dehydrogenase-like protein YdjJ from *E. coli* (wheat, 5vm2), and scyllo-inosose 3-dehydrogenase from *Thermotoga maritima* (3IP1, yellow)

In order to gain a deeper understanding of the molecular function, we performed X-ray crystallography studies of FoADH and ZoADH. For the determination of the functional states of both ADHs, the essential NAD^+^ cofactor was added to purified FoADH and ZoADH proteins before crystallization. The crystal structures of FoADH and ZoADH in complex with NAD^+^ were determined at a resolution of 2.5 and 2.1 Å, respectively (Table S2). FoADH and ZoADH crystals belong to the space group monoclinic P2_1_ and orthorhombic P2_1_2_1_2_1_, respectively, and contain four and eight molecules in the asymmetric unit, respectively (Fig. S10). The electron density map of FoADH and ZoADH clearly showed the almost entire polypeptide chain, except for a partially disordered fragment of the loop between the β5- and β6-strands (Gly111-His115 in both enzymes), which is involved in substrate binding and specificity. The monomer structures of FoADH and ZoADH comprise the catalytic domain (residues 1–149 and residues 283–326 for both enzymes) and the cofactor-binding domain (residues 150–282 for both enzymes) (Fig. 6a), which are separated by a cleft containing a deep pocket, which accommodates the substrate and the NAD^+^ cofactor. The catalytic domain contains two zinc-binding sites, Zn1 and Zn2, which are responsible for catalytic activity and structural stability, respectively. The cofactor binding domain adopts a typical Rossmann fold with the conserved sequence “GXGXXG.” FoADH and ZoADH had a 76.0% similarity in amino acid sequence (Fig. S11), and their monomer structures showed a similarity with a root-mean-square deviation (r.m.s.d.) of 0.350–0.772 Å (Table S4).

In FoADH, molecules A/B/C/D and E/F/G/H form a tetrameric formation (Fig. S10). In the superimposition of monomeric FoADH molecules, the A, B, C, E, and G molecules showed structural similarity (denoted as closed form) with a r.m.s.d. of 0.256–0.353 Å, whereas molecules D and H (denoted as open form) showed the relatively high r.m.s.d. value of 0.457–0.626 Å when superimposed with molecules A, B, C, E, and G (Fig. 6b; Table S5). On the other hand, molecule F maintains the intermediate conformation between the closed and open conformations. When the cofactor binding domains of molecules A and H of FoADH were superimposed, the catalytic binding of molecule H was shifted by approximately 2.0–3.3 Å in the opposite direction of the substrate-binding cleft compared to molecule A (Fig. 6b).

In ZoADH, the superimposition of molecules A, B, and C exhibited a similar conformation (denoted as closed form) with a r.m.s.d. of 0.198–0.226 Å, whereas molecule D (denoted as open form) showed a relatively high r.m.s.d. value of 0.314–0.471 Å when superimposed with molecules A, B, and C (Fig. 6b; Table S6). Superposition of the cofactor binding domains of molecules A and D clearly revealed the conformational difference between the catalytic domains. The catalytic domain of molecule D is shifted about 2.2–3.3 Å to the outside of the substrate binding cleft of ZoADH compared to molecule A. Accordingly, in the structure of NAD^+^-bound FoADH, molecules A/B/C and D represent closed and open conformations of the substrate binding site, respectively. Collectively, the crystal structures of NAD^+^-bound ZoADH and FoADH contain open and closed conformations between catalytic and cofactor-binding domains (see below).

The crystal structures of FoADH and ZoADH showed the tetrameric formation via the arrangement of a dimer of dimers (Fig. 6c). In both ADHs, the β17- and β18-strands of the cofactor binding domains are stabilized by forming an antiparallel β-sheet with the β17* and β18* strands (asterisk indicates the second monomer), respectively (Figs. S12 and S13). For FoADH, the dimeric interface is stabilized by the main chain interactions of Ile297-Ile299* (asterisk denotes the partner molecule) and Ile299-Ile297* between the β17 strands and Tyr310-Tyr310* between β18 strands (Fig. S12). In addition, numerous hydrogen and salt bridges were observed in the dimer interface with a buried surface area of 1654 Å^2^ (Table S7). The dimer of dimers is stabilized by hydrogen interaction and the buried interface of dimers of dimers is 1193 Å^2^ (Table S7). For ZoADH, the dimeric interface is stabilized by the main chain interactions of Ile298-Ile300* and Ile300-Ile298* between the β17 strand and Tyr311-Tyr311* between the β18 strand (Fig. S13). Moreover, numerous hydrogen and salt bridges were observed at the dimer interface with a buried surface area of 1640 Å^2^ (Table S8). The dimer of dimers is stabilized by hydrogen interactions and salt bridges and the buried interface of dimers of dimers is ~ 1205 Å^2^ (Table S8). All active sites of the tetrameric ADH in the crystal were exposed to solvent (Fig. 6c). Superposition of tetrameric molecules of FoADH and ZoADH in the asymmetric unit shows a r.m.s.d. of 0.327–0.888 Å for whole Cα atoms (Fig. 6d).

Structural homology search by DALI revealed that both FoADH and ZoADH share structural similarities to the class II alcohol dehydrogenase (ADH4) from humans (PDB code: 3COS, Z-score = 45.8 for FoADH and 45.3 for ZoADH, sequence identity = 32% for FoADH [357α atoms] and 30% for ZoADH [357α atoms]), an ADH from *E. coli* (PDB code: 5vm2, Z-score = 48.1 for FoADH and 38.1 for ZoADH, sequence identity = 28% for FoADH [329α atoms] and 27% for ZoADH [328α atoms]) as well as an ADH from *Thermotoga maritima* (PDB code: 3IP1, Z-score = 35.8 for FoADH and 36.8 for ZoADH, sequence identity = 25% for FoADH [328α atoms] and 23% for ZoADH [332α atoms]). Although these structural homologous ADHs share low amino acid sequence similarities with less than 32% compared to FoADH and ZoADH, the active site residues involved in the Zn^2+^ and NAD^+^ binding are highly conserved (Fig. S11). In addition, the NAD^+^-binding domain exhibits a typical Rossmann fold motif and has the classical conserved sequence “GXGXXG” as in other ADHs, and the topologies of those ADHs are highly similar (Fig. S11). The overall topology of those homolog structures was similar to FoADH and ZoADH (Fig. S14). However, superimposition of those ADH structures revealed that there is a large difference in conformation between catalytic and cofactor-binding domains with a r.m.s.d. of 1.373–2.963 Å for FoADH and 1.376–2.191 for ZoADH (Fig. 6e), indicating that they possess large distinct NAD^+^ and substrate-binding clefts. Meanwhile, ADHs from *E. coli* and *T. maritima* also formed the tetrameric formation in crystal structures like FoADH and ZoADH (Fig. S14). These ADHs have a similar tetrameric assembly; however, the superimposition of the tetrameric ADHs showed that these tetrameric assemble have low similarity with a r.m.s.d. of 17.68 ~ 29.94 Å.

### NAD^+^ and Zn^2+^-binding sites of FoADH and ZoADH

**Fig. 7:**
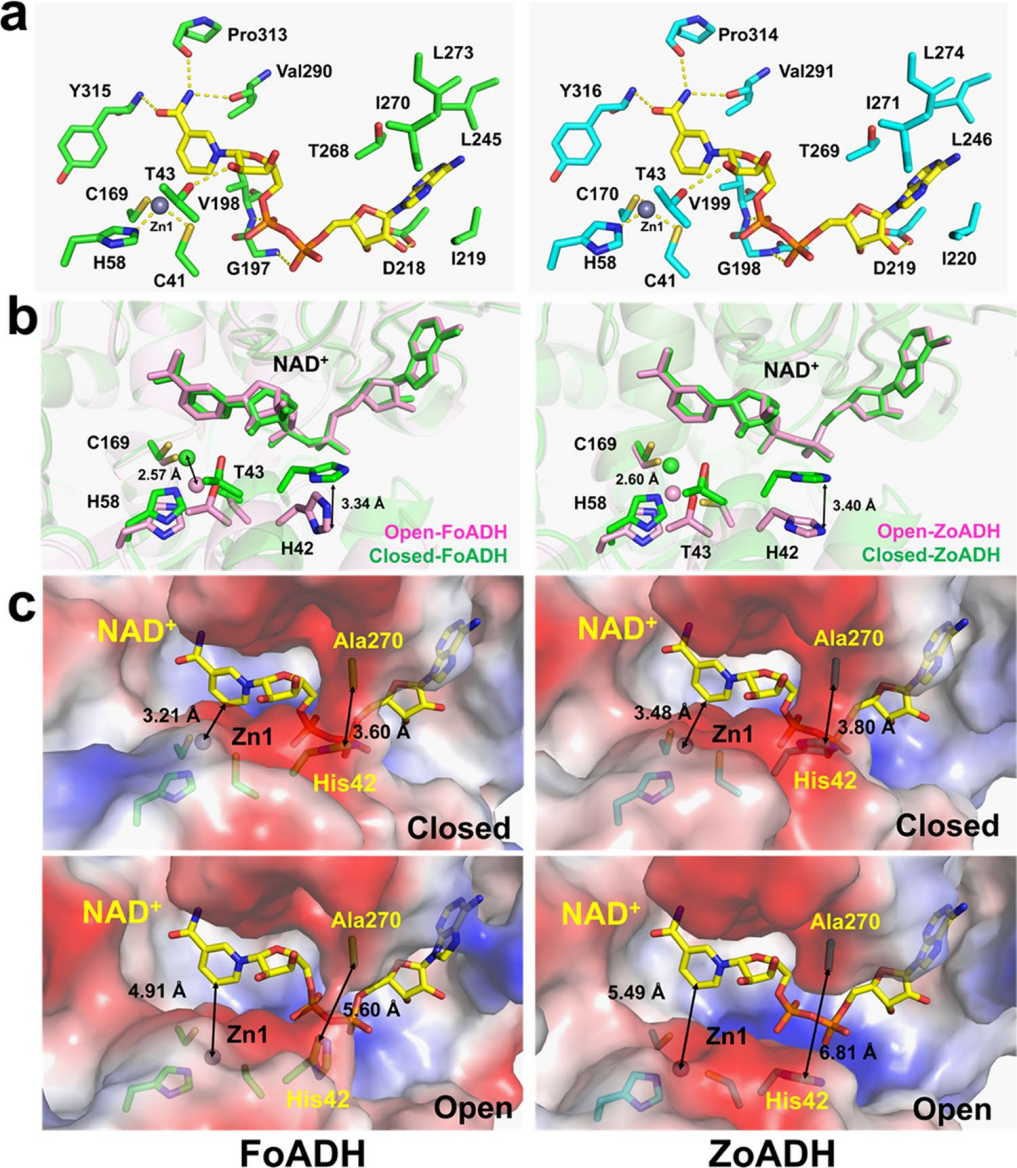
Active sites of FoADH and ZoADH. **a** Interaction of ZoADH and FoADH with NAD^+^ and zinc ions at the Zn1 site. **b** Superimposition of the active sites of open and closed conformations of FoADH and ZoADH. **c** Comparison of electrostatic surface structures of open and closed conformations of FoADH and ZoADH

While NAD^+^ is the required cofactor for alcohol oxidation, Zn^2+^ interacts with the alcohol molecule in the active site. The electron density maps of a NAD^+^ molecule and two zinc ions are clearly observed in a substrate-binding cleft of both FoADH and ZoADH (Fig. S15). The binding configuration of NAD^+^ and the Zn^2+^ ions of ZoADH and FoADH are highly similar (Fig. 7a). The adenine ring of NAD^+^ is located in the hydrophobic pocket formed by hydrophobic interaction (Ile219, Leu245, Thr268, Ile270, and Leu273 for FoADH, Ile220, Leu246, Thr269, Ile271, and Leu274 for ZoADH). The adenine ribose appears to be in a C2’-endo conformation, and the O2’ and O3’-hydroxyl group of ribose forms a hydrogen bond with the side chain of aspartate (Asp218 for FoADH and Asp219 for ZoADH). The pyrophosphate moiety of the NAD^+^ interacts with the nitrogen atoms of the main chain of glycine-valine residue (Gly197-Val198 for FoADH and Gly198 and Val199 for ZoADH) that forms the loop between strand β5 and helix α4. The nicotinamide ribose is in a C2’-endo conformation, and hydrogen bonds are formed between the ribose O2’-hydroxyl group and threonine (Thr43 for FoADH and ZoADH). The nicotinamide ring is in the anti-conformation. The carboxamide nitrogen atom of the nicotinamide ring interacted with the main chain of proline (Pro313 for FoADH and Pro314 for ZoADH) and valine (Val290 for FoADH and Val291 for ZoADH). The carboxamide oxygen atom of the nicotinamide ring interacted with the main chain of tyrosine (Tyr315 for FoADH and Tyr316 for ZoADH). Therefore, in both FoADH and ZoADH, the NAD^+^ molecules are stabilized by hydrophobic and hydrogen bond interactions.

In both FoADH and ZoADH, two zinc ions are commonly observed in the active site (Zn1 site) and in a loop between α2 and β7 (Zn2 site) (Fig. 7a; Fig. S15). The zinc ion at the Zn1 site is coordinated by conserved cysteine and histidine residues (Cys41, His58, and Cys169 for FoADH and Cys41, His58, and Cys170 for FoADH) in the catalytic domain. The zinc ion at the Zn2 site is involved in the protein stability and is tetrahedrally coordinated by conserved cysteine residues (Cys88, Cys91, Cys94, and Cys102 for both enzymes) (Fig. S15). Their result indicated that ZoADH and FoADH showed high structural similarity for the NAD^+^ and zinc-binding configuration.

Different structural conformations were observed between monomeric ADHs in the tetrameric formation of FoADH and ZoADH (Fig. 6b), indicating that they exhibit structurally different substrate binding clefts and active sites. In both results of superimposition of the active sites of FoADH and ZoADH, the positions of the NAD^+^ and Zn2 sites were similar, whereas a significant difference was observed in the positions of the catalytic Zn1 sites (Fig. 7b). In FoADH and ZoADH, the maximum distances between metals from the Zn1 site were 2.57 and 2.60 Å, respectively, from the closed and open conformations of two domains of ADHs (Fig. 7b).

Since the substrate binds to the Zn1 site and a dehydrogenase reaction occurs through the interaction of NAD^+^ with the hydroxyl group, the size of the space between NAD^+^ and Zn1 is involved in substrate selectivity. The closest/longest distances between the Zn^2+^ and C5 atoms of the nicotinamide ring of NAD^+^ in FoADH and ZoADH were 3.21/4.91 Å and 3.46/5.49 Å, respectively (Fig. 7c). These different distances between Zn^2+^ and NAD^+^ were caused by the different closed and open conformations of FoADH and ZoADH.

The electrostatic surfaces of FoADH and ZoADH showed that the substrate binding sites commonly exhibited a hydrophobic surface (Fig. 7c). The space of the substrate binding site of FoADH in closed and open conformations was approximately 3.4 × 4.2 Å and 3.9 × 5.4 Å, respectively (Fig. S15). In the closed and open conformations of FoADH, His42 and Ala270 are apart by 3.60 and 5.60 Å, respectively, showing the surface structures surrounding the NAD^+^ (Fig. 7c). ZoADH also exhibits open and closed conformations similar to FoADH, but the distance of open conformation is relatively wide. The space of the substrate binding site of ZoADH in closed and open conformation was approximately 3.0 × 3.8 Å and 3.8 × 4.9 Å, respectively (Fig. S15). In the closed conformation of ZoADH, the catalytic domain and the cofactor domain are close to each other, especially His42 and Ala270 by a distance of 3.88 Å, indicating the surface structure surrounding the NAD^+^ (Fig. 7c). On the other hand, in the open conformation of ZoADH, His42 and Ala270 are apart by 6.81 Å, and accordingly, the entire NAD^+^ molecule in the surface structure is exposed to the solvent (Fig. 7c).

## Discussion

In the present work, FoADH from *F. agariphila* KMM 3901^T^ and ZoADH from *Z. galactanivorans* Dsij^T^ were characterized in detail to draw conclusions about their biological function. Three main conclusions regarding biological function can be derived from the knockout of the genes encoding for ZoADH and CYP in *Z. galactanivorans* and subsequent growth studies on d-galactose and G6Me. First, we confirmed the hypothesis of Reisky et al. that in the absence of CYP-catalyzed oxidative demethylation, a G6Me utilization as the sole carbon source is infeasible for the organism (Reisky et al. 2018). Surprisingly, the knockout of the ZoADH gene also caused diminished growth of *Z. galactanivorans* in the presence of G6Me. Second, due to this observation, we can conclude a significant role of these ADHs in G6Me utilization in these marine bacteria. From an ecological perspective, this has additional importance for marine carbohydrate degraders. G6Me can occur up to 28% within the porphyran chain (Rees and Conway 1962). Thus, reduced utilization of G6Me would represent a substantial potential loss as a carbon source for the organism. Third, since normal growth was observed in the presence of d-galactose as the sole carbon source, a function in d-galactose metabolism can be excluded. This was also supported by the observation that both ADHs lacked activity for d-galactose. The ADHs are therefore probably involved in oxidative demethylation or a subsequent reaction. Since no activity was observed for G6Me, the substrate of oxidative demethylation could be excluded. Consequently, we hypothesized that the ADHs are involved in the detoxification of formaldehyde, which is a by-product of the oxidative demethylation reaction. This was also supported by the resistance of both ADHs to formaldehyde exposure. Formaldehyde is a toxic metabolite due to its properties as a highly reactive electrophile. It can react with free amino and thiol groups of proteins and nucleic acids, leading to protein and DNA damage as well as cross-link formations (Chen et al. 2016; Shishodia et al. 2018; Tayri-Wilk et al. 2020). It has been shown that higher concentrations of formaldehyde can negatively affect the growth of *Z. galactanivorans* (Brott et al. 2022). Thus, reduced growth of the ADH knockout strain could be explained by the potential accumulation of formaldehyde. There are numerous metabolic pathways in which formaldehyde can be detoxified (Yurimoto et al. 2005; Klein et al. 2022). However, in the thiol-dependent formaldehyde detoxification, a zinc-dependent ADH and an esterase perform the key reactions (Sanghani et al. 2000; Gonzalez et al. 2006). Genome neighborhood analysis revealed that most marine bacteria that possess the ADH gene are located in close proximity to a gene encoding for an esterase in addition to the CYP gene. We, therefore, investigated whether the ADH catalyzed thiol-dependent detoxification of formaldehyde. However, with glutathione, mycothiol, and bacillithiol as thiol cofactors, no activity was detected for either ADH. These observations can be further explained by the crystal structures of both ADHs; sterically demanding compounds such as mycothiol or bacillithiol cannot fit into the narrow active site of these enzymes. These observations are also consistent with the results from the sequence similarity network, in which glutathione- and mycothiol-dependent formaldehyde dehydrogenases were predominantly present in different clusters (main clusters 1 and 4) than the ADHs (main cluster 2). Since no activity could be detected with literature-known cofactors, additional thiols were considered; however, no activity could be observed either. Thiol cofactors are still being discovered (Newton and Rawat 2019); perhaps marine organisms also possess an unidentified thiol, which can serve as a cofactor for this reaction. Since no activity was observed for formaldehyde without an additional thiol cofactor, the biological function of a thiol-independent formaldehyde dehydrogenase was excluded. In addition, some ADHs can possess dismutase activities (Trivić et al. 1999). A formaldehyde dismutase catalyzes the disproportionation of formaldehyde to methanol and formic acid in the presence of a covalently bound NAD^+^ (Yonemitsu and Kikuchi 2018). However, this reaction could not be detected. Both organisms harbor other metabolic pathways for the detoxification of formaldehyde (Brott et al. 2022). For instance, in *Z. galactanivorans*, the genes encoding the key enzymes of the ribulose monophosphate pathway are upregulated in the presence of porphyran (Brott et al. 2022), so an accumulation of formaldehyde is unlikely. Eventually, the ADHs might have a completely different biological function, such as the regeneration of NADH (Hilberath et al. 2021; Kokorin et al. 2021). In the oxidative demethylation reaction, NADH is oxidized to NAD^+^, and a reduced growth in the ADH knockout strain due to cofactor depletion might be possible. NADH could be regenerated by the oxidation of an unknown component or by the thiol-dependent formaldehyde detoxification pathway. However, it is doubtful that the loss of one single enzyme would cause such a tremendous effect on NADH/NAD^+^ homeostasis. Additionally, the ADHs displayed predominantly activity for the reduction of aldehydes under NADH consumption, so recycling of a cofactor is improbable.

Both ADHs possessed predominantly activity for aromatic substances, resulting in a substrate specificity resembling partially those of cinnamyl alcohol and/or benzyl alcohol dehydrogenases (Larroy et al. 2002; Willson et al. 2022). However, the highest activity was observed for pyridine-3-carbaldehyde and furan derivatives. Furfural is generally produced as a side product by pretreating lignocellulosic biomass for the production of bioethanol. Under acidic conditions and high temperatures, dehydration of pentoses and hexoses proceeds, leading to the formation of furfural or hydroxymethylfurfural. Furfural acts as an inhibitor in subsequent bioethanol-producing fermentations by bacteria by prolonging the lag phase of growth and thereby the fermentation time (Mariscal et al. 2016). Consequently, these marine bacteria possess ADHs that catalyze the potential removal of furfural, although the biological function may be different. The ADHs lacked activity for various sugar substrates, which excluded a polyol dehydrogenase activity. Activity for any other monosaccharides, disaccharides, or even oligosaccharides formed during porphyran degradation is unlikely as well, considering the substrate specificity of the enzymes based on the narrow active site. The data from biochemical characterizations are discussed in the SI.

We have determined the crystal structures of FoADH and ZoADH complexed with NAD^+^ and two zinc ions. These ADHs showed high structural similarity in terms of topology and assembly. On the one hand, these two ADHs showed similarities in topology with other ADHs from humans, *E. coli*, and *T. maritima*, but showed distinct conformation between the cofactor and catalytic domains of those ADHs. On the other hand, the crystal structures of FoADH and ZoADH showed open and closed conformations, indicating that the conformation between the two domains can change in the state where the substrate is not bound. These distinct conformations of FoADH and ZoADH represent different substrate binding pockets. When they exhibit an open conformation between the two domains of FoADH and ZoADH, they form a broadened substrate-binding pocket. Accordingly, in terms of substrate accessibility, we consider that substrate accessibility will be easier when FoADH and ZoADH have an open conformation.

During substrate recognition, when the converting functional group from the substrate approaches the Zn1 site on the substrate binding pocket of FoADH and ZoADH, the rest of the substrate is exposed to the nicotinamide of NAD^+^ or the hydrophobic surface. Considering that the nicotinamide group of NAD^+^ is involved in the oxidoreductase mechanism of the ADH, the substrate would prefer to be located on the hydrophobic surface rather than the nicotinamide group of NAD^+^. Accordingly, FoADH and ZoADH may prefer substrates having a hydrophobic body. Our biochemical studies showed that both enzymes prefer aromatic substrates. We expected that the aromatic ring of the substrate may be located on a hydrophobic surface nearby the substrate binding pocket of FoADH and ZoADH. In this case, the aromatic ring of the substrate could interact with the Phe136 residue in the hydrophobic surfaces of the enzymes. Based on the active site structures of both, ADH computational docking of a substrate will be able to provide an insight into the molecular mechanism and substrate specificity. However, from the results of this study, ZoADH and FoADH have various conformations between catalytic and cofactor binding domains in NAD^+^ and two zinc ion-binding states, indicating the computational docking results could be different depending on the applied model structure. Also, based on our results, we concluded that the docking results may be different from biochemical experiments if the active sites of ZoADH and FoADH may have different conformations. Therefore, to better understand the substrate specificity, the crystal structures of ZoADH and FoADH in complex with the biological substrate will be needed in the future.

In summary, in this study, we determined the putative functions of conserved ADH from marine *Flavobacteriia*. Additionally, we provided the crystal structures of the enzymes of* F*. *agariphila* and *Z. galactanivorans*. Enzymatic studies revealed the preferential conversion of aromatic aldehydes. We revealed that these enzymes are not involved in formaldehyde detoxification or in the subsequent reaction of the oxidative demethylation of G6Me. Based on gene knockouts, we demonstrated the essential role of these ADHs in the utilization of marine algal sugars. Our study indicates a potential auxiliary activity of these ADHs in the utilization of algal sugars by marine *Flavobacteriia*.

## Supplementary Information

Below is the link to the electronic supplementary material.

Supplementary file1 (PDF 15930 kb)

## Data Availability

The datasets generated during and/or analyzed during the current study are available from the corresponding author upon reasonable request.
