# Supplementary material for: Unique alcohol dehydrogenases involved in algal sugar utilization by marine bacteria
Source: Appl Microbiol Biotechnol. 2023 Mar 7;107(7-8):2363–84. doi: 10.1007/s00253-023-12447-x (PMC10033563; doi:10.1007/s00253-023-12447-x)
Supplement: Supplementary file 1 — Supplementary file1 (PDF 15930 kb) [file 253_2023_12447_MOESM1_ESM.pdf]

Supporting Information

Unique alcohol dehydrogenases involved in algal sugar utilization by marine bacteria

Stefan Brott[1], Ki Hyun Nam[2], François Thomas[3], Theresa Dutschei[1], Lukas Reisky[1], Maike Behrens[1], Hanna C. Grimm[1], Gurvan Michel[3], Thomas Schweder[4], and Uwe T. Bornscheuer[1]*

[1]Department of Biotechnology & Enzyme Catalysis, Institute of Biochemistry, University of Greifswald, Greifswald 17487, Germany

[2]Department of Life Science, Pohang University of Science and Technology, Pohang 37673, South Korea

[3]Laboratory of Integrative Biology of Marine Models (LBI2M), Station Biologique de Roscoff (SBR), Sorbonne Université, CNRS 29688 Roscoff, Bretagne, France

[4]Department of Pharmaceutical Biotechnology, Institute of Pharmacy, University of Greifswald, Greifswald 17487, Germany

*Corresponding author: E-mail: uwe.bornscheuer@uni-greifswald.de

Table of Contents

Additional Discussion……………………………………………………………………………3

Figures

Fig. S1 Sequence similarity network from FoADH and ZoADH……………………………....4

Fig. S2 Genome neighborhood diagram of main cluster 2……………………………………....5

Fig. S3 SDS-PAGE of purified proteins………………………………………………………….6

Fig. S4 Effect of NaCl on enzyme activity……………………………………………………….6

Fig. S5 Influence of different metal ions on enzyme activity…………………………………….7

Fig. S6 Influence of EDTA, DTT and 2-Mercaptoethanol on enzyme activity………………….7

Fig. S7 Influence of water-miscible solvents on enzyme activity……………………………….8

Fig. S8 Influence of formaldehyde on enzyme activity……………………………………….....8

Fig. S9 Asymmetric units of both ADHs……………………………………………………….....9

Fig. S10 Structure-based amino acid alignment of both ADHs…………………………………10

Fig. S11 Dimeric interface of both ADH…………………………………………………………11

Fig. S12 Structural comparison of ZoADH and FoADH with structural homologs……………12

Fig. S13 Electron density maps…………………………………………………………………13

Fig. S14 Substrate binding pocket of FoADH and ZoADH……………………………………14

Tables

Table S1 Primers used for knockout experiments………………………………………….......15

Table S2 Data collection and refinement statistics…………………………………………..….16

Table S3 The ADHs lacked activity for various sugar substrates…………………………......17

Table S4 Superimposition of monomer structures of FoADH and ZoADH………………........18

Table S5 Superimposition of monomeric structures of FoADH…………………………….......18

Table S6 Superimposition of monomeric structures of ZoADH…………………………….......18

Table S7 Hydrogen bonds and salt bridges on the interface of FoADH……………………..….19

Table S8 Hydrogen bonds and salt bridges on the interface of ZoADH……………………......23

References.……………………………………………………………………………………25

Additional Discussion

The biochemical characteristics of both enzymes are almost identical. The pH optimum for the reduction and oxidation reactions are different, similar observations have also been reported for several other ADHs (Ying et al. 2014; Akal et al. 2019; Zhang et al. 2021). The optima in *Z. galactanivorans* also represent the pH range in which this organism prefers to grow (Barbeyron et al. 2001). Increased activity for the oxidation reaction was observed for both ADHs in the presence of Tris-HCl buffer. Most likely, this is caused by a potential subsequent reaction between formed aldehyde and the primary amine of Tris, which might shift the equilibrium towards product formation. Reactions between aldehydes and Tris are well known in literature (Bubb et al. 1995) and may possess an influence on the enzyme activity of ADHs (Trivić et al. 1998). In contrast, almost no activity was detected in the presence of borate buffer; similar observations were reported for an ADH from yeast, where borate performed a competitive inhibition with respect to NAD^+ (Smith and Johnson 1975).

The temperature optima of both enzymes differ dramatically from the preferred growth temperatures of *F. agariphila* with 23 °C (Nedashkovskaya et al. 2006) and *Z. galactanivorans* with 35 °C (Barbeyron et al. 2001). Both bacteria engage in algae-associated life and tend to inhabit shallow water, which implies that such high temperatures are unexpected under natural conditions. Therefore, the temperature optimum is probably a result of the thermal stability of both enzymes caused by the stabilizing effects of the Zn2 site and the numerous hydrogen and salt bridges. Simultaneously, these structural properties ensure stability at temperatures encountered in the habitat of these marine bacteria.

A slight increase and a subsequent reduction in the activity of both enzymes was observed with increasing salt concentration. Both enzymes originate from a marine environment where a salinity of 3.5% (w/v), corresponding to approximately 600 mM NaCl, exists. At this concentration a negative effect on activity was observable. However, coastal areas where algal blooms tend to occur more frequently can also contain a reduced salinity due to freshwater influxes. In addition, a different salt concentration might be present within the cell. Considering that the ADHs possess an intracellular function, it might be reasonable to assume that the ADHs are not exposed to higher salt concentrations, which would also explain the beneficial effect at a lower NaCl concentration.

Incubation with EDTA and the resulting partial loss of enzyme activity demonstrated that the zinc ion located in the active site is required for the ADH activity. However, since no complete inhibition of ADH by EDTA could be obtained, some resistance to chelation can be assumed. Inhibition of both ADHs was observed in the presence of additional zinc, similar findings were described for the allyl/benzyl alcohol dehydrogenase from *Yokenella sp.* (Ying et al. 2014) and

the aryl alcohol dehydrogenase from *Acinetobacter baylyi* (Uthoff and Steinbüchel 2012). Unexpectedly, a 10-14-fold increase in relative activity was observed in the presence of Mn^{2+}, Ni^{2+} and Co^{2+}. Finding an explanation for these phenomena is challenging, perhaps one of these ions is the natural metal cofactor of these ADHs.

Figures

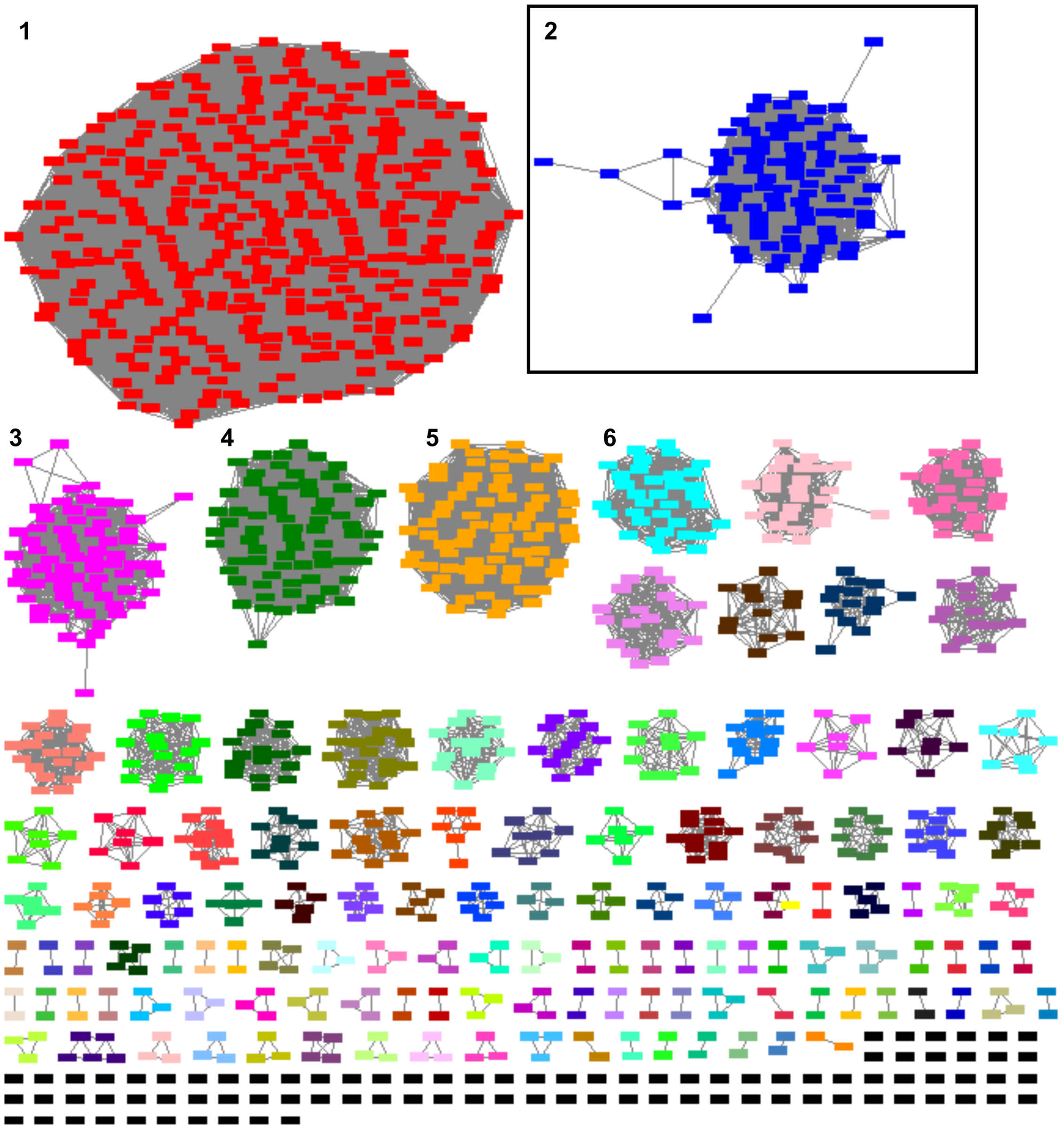

Fig. S1 Sequence similarity network from FoADH and ZoADH. Both ADHs cluster with zinc-dependent ADHs and some glutathione-dependent formaldehyde dehydrogenases. Sequence similarity network which were obtained as a result from the FoADH and ZoADH blast against MarDB and MarRef sequences (alignment score 150, 63.14%ident). Overall, six main clusters (1-6) were obtained, a main cluster is defined here as a cluster that contains at least 34 sequences, with FoADH und ZoADH included in main cluster 2 (framed).

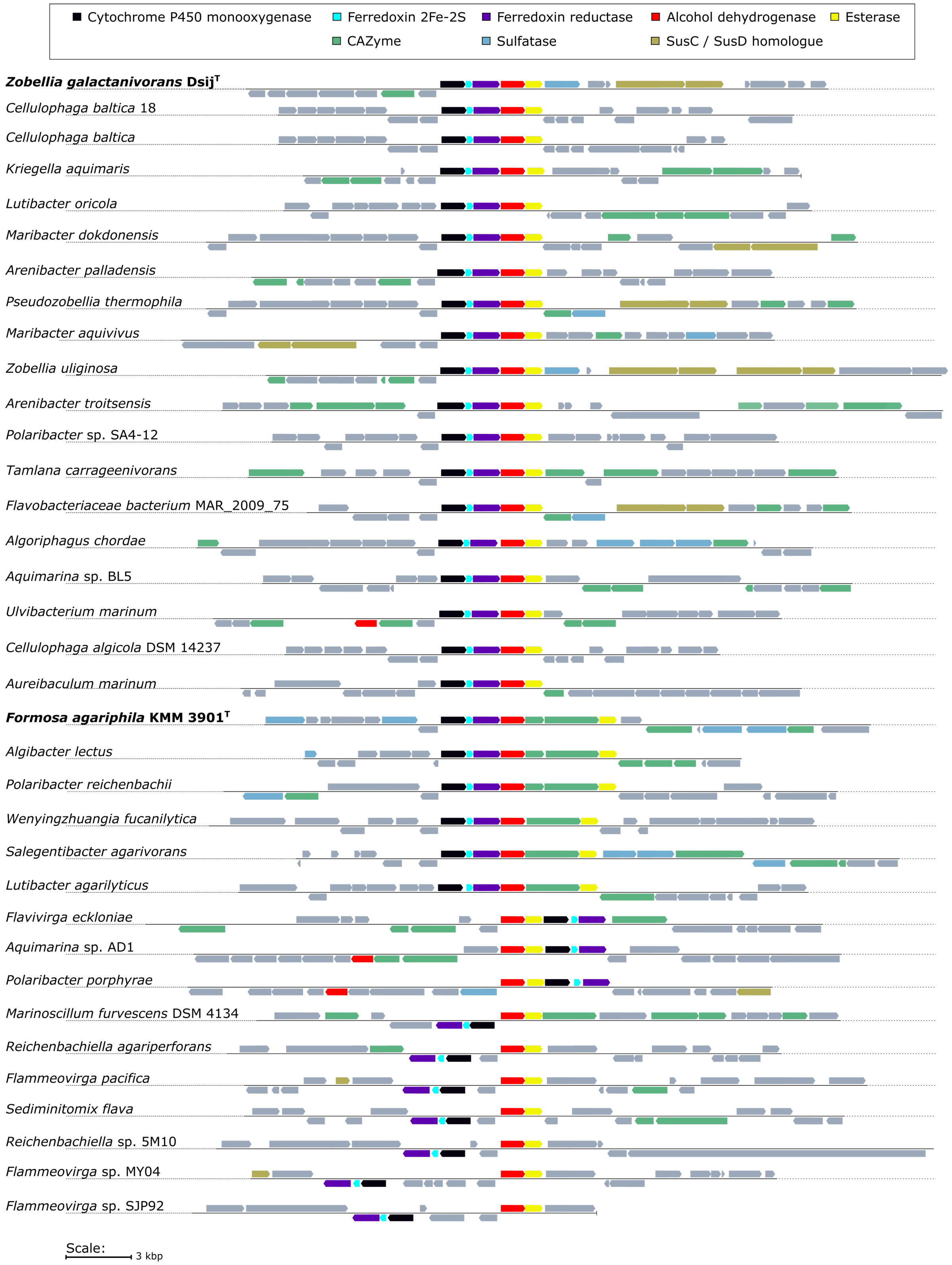

Fig. S2 Genome neighborhood diagram of main cluster 2. The main cluster 2 of the sequence similarity network (Fig. S1) reveals that the gene encoding for the ADH is consistently in close proximity to the genes encoding for the enzymes of oxidative demethylation. Simultaneously, the analysis reveals that these ADHs are represented in several marine carbohydrate utilizers. In order to achieve a better overview, other genes not involved in carbohydrate degradation or uptake/binding have been grayed out.

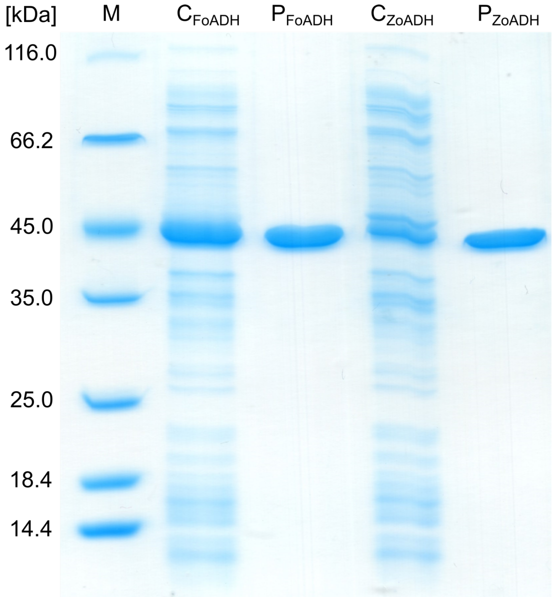

Fig. S3 SDS-PAGE of purified proteins. The purified proteins (P) and the crude cell extract (C) were separated on a 12.5% gel and stained with Coomassie blue. 4.0 µg of the proteins were loaded onto the gel. As reference (M) the Pierce™ Unstained protein molecular weight marker (Thermo Scientific, Waltham, MA, USA) was used. Both enzymes possess a theoretical molecular weight of approximately 40.9 kDa. The experiment was repeated independently with similar results.

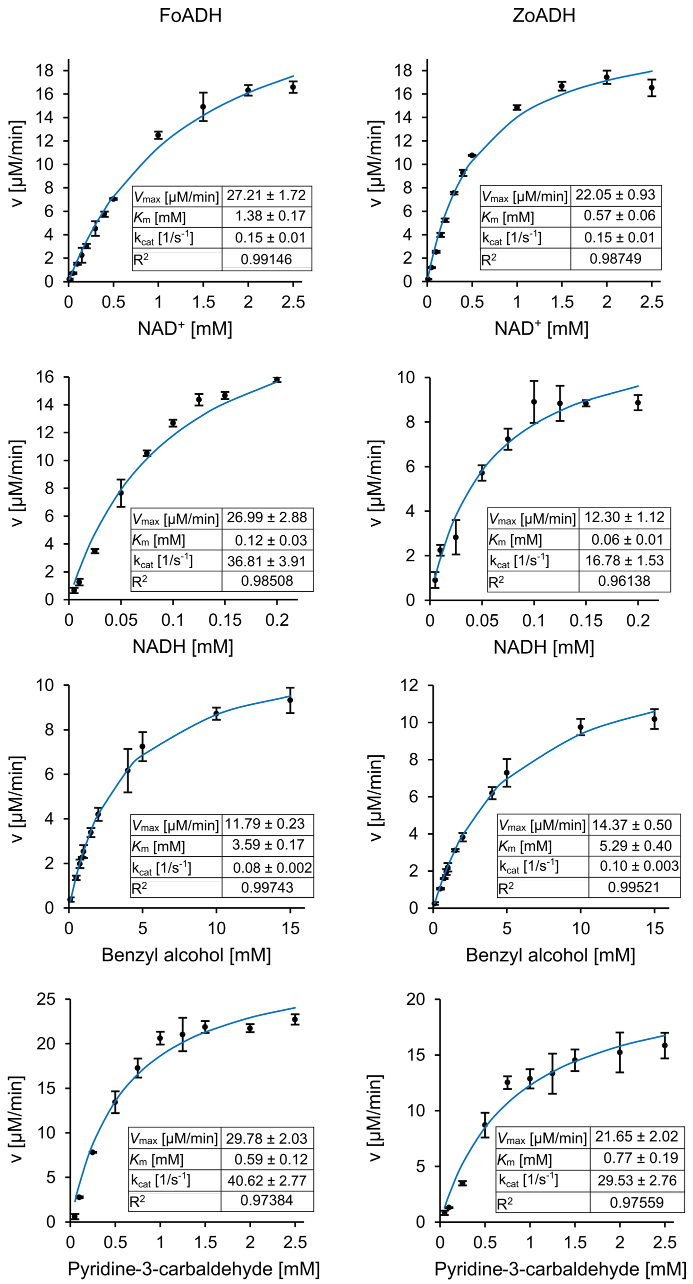

Fig. S4 Kinetic data for FoADH and ZoADH. The final protein content employed for the oxidation reactions was 0.1 mg mL^{-1}, which corresponds to a protein concentration of 2.44 µM. For the determination of the kinetic parameters for NAD^+, 15 mM benzyl alcohol was used as the final substrate concentration, while a final cofactor concentration of 5 mM NAD^+ was used for the determination of the kinetic parameters for benzyl alcohol. Oxidation reactions were performed in 50 mM NaPi buffer pH 8.5 and at a reaction temperature of 70 °C. In the reduction reaction, the final protein content used was 5 µg mL^{-1}, which is equivalent to a protein concentration of 0.012 µM. For the determination of the kinetic parameters for NADH, 2.5 mM pyridine-3-carbaldehyde was used as the final substrate concentration, while a final cofactor concentration of 0.5 mM NADH was used for the determination of the kinetic parameters for pyridine-3-carbaldehyde. Reduction reactions were carried out in 50 mM succinate buffer pH 6.5 and at 70 °C. All measurements were carried out as triplicates, the mean values (black dots) and their standard deviations are given. V_{max} and K_m were determined via a fitted curve (blue line), which was calculated using standard Michaelis-Menten kinetics.

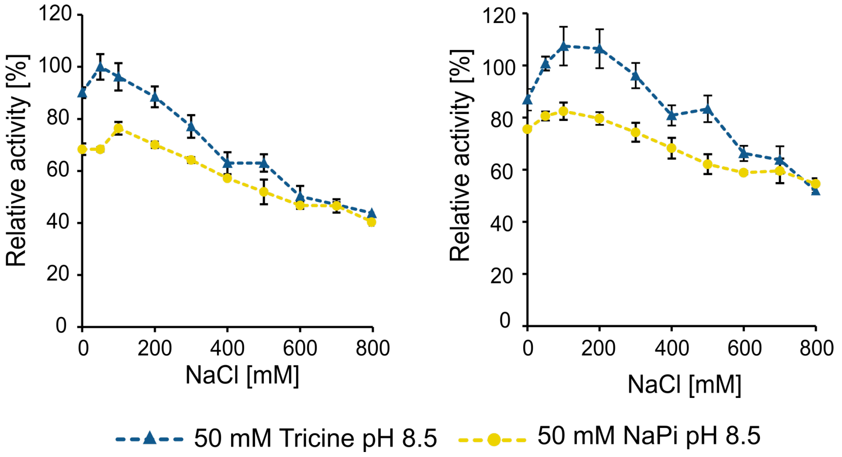

Fig. S5 Effect of NaCl on enzyme activity. Determination of NaCl influence on enzyme activity was performed by carrying out the oxidation reaction in the presence of different NaCl concentrations varying from 0 to 800 mM. The maximum relative activity (100%) corresponds to the measurement at a NaCl concentration of 50mM for FoADH and 100 mM for ZoADH in the 50 mM Tricine buffer. Assay conditions were as follows: the reaction volume was 200 µL, 10 mM benzyl alcohol was used as substrate, the final enzyme concentration was 0.1 mg mL^{-1}. The reaction was carried out at 25 °C in a 50 mM NaPi buffer pH 8.5 or in a 50 mM Tricine buffer pH 8.5 and started by the addition of 0.5 mM NAD^{+}. All measurements were performed as triplicates, the mean is given and the error bars indicate the standard deviation.

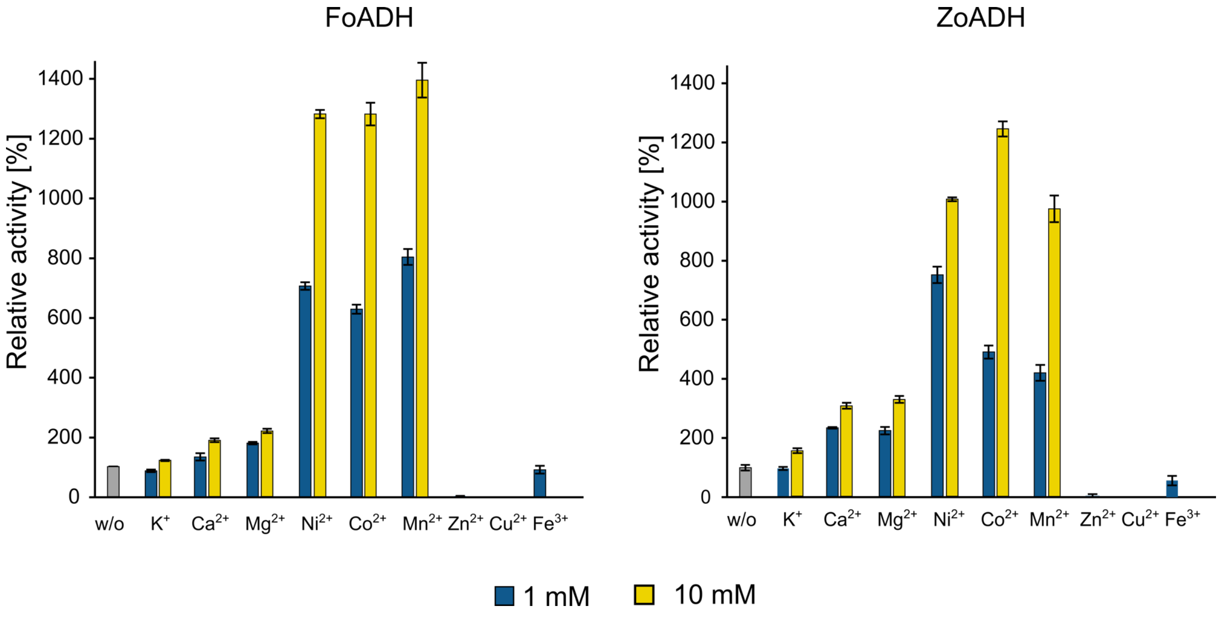

Fig. S6 Influence of different metal ions on enzyme activity. The ADH with was incubated with the metal chloride for 1 h at RT prior to measurement. The maximum relative activity (100%) corresponds to the measurement for the control, which was incubated without a metal ion. The measurement was performed under following conditions: a final substrate concentration of 10 mM benzyl alcohol and 0.5 mM NAD+ was used. The reaction was started by the addition of ADH at a final enzyme concentration of 0.1 mg mL^{-1}. The measurement was performed in a 50 mM HEPES buffer pH 8.5 at 25 °C. All measurements were performed as triplicates, the mean and the standard deviation is given.

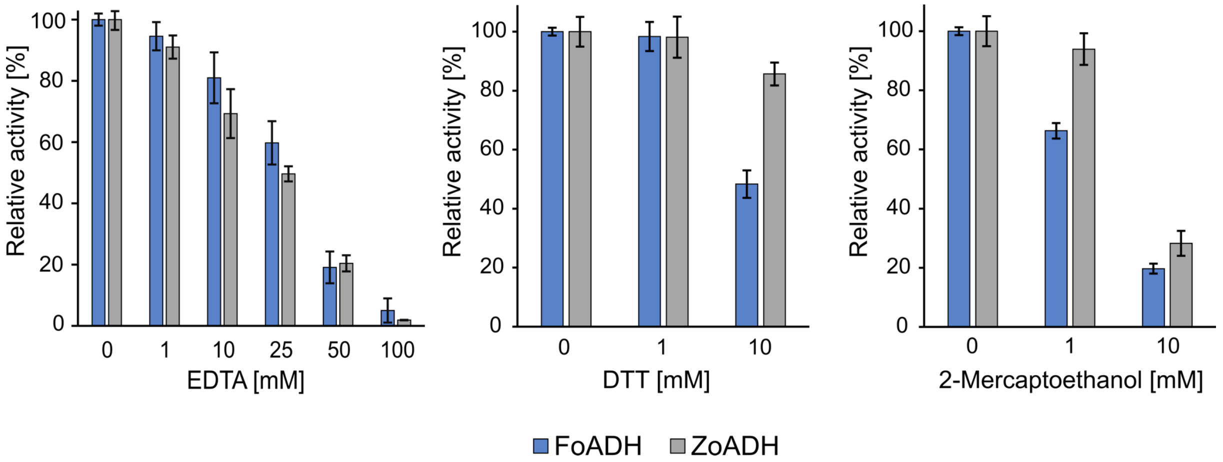

Fig. S7 Influence of EDTA, DTT and 2-Mercaptoethanol on enzyme activity. The ADH was incubated with the compound for 1 h at RT prior to measurement. The maximum relative activity (100%) corresponds to the measurement for the control, which was incubated without additional compound. The measurement was performed under following conditions: a final substrate concentration of 10 mM benzyl alcohol and 0.5 mM NAD+ was used. The reaction was started by the addition of ADH at a final enzyme concentration of 0.1 mg mL^{-1}. The measurement was performed in a 50 mM HEPES buffer pH 8.5 at 25 °C. All measurements were performed as triplicates, the mean and the standard deviation is given.

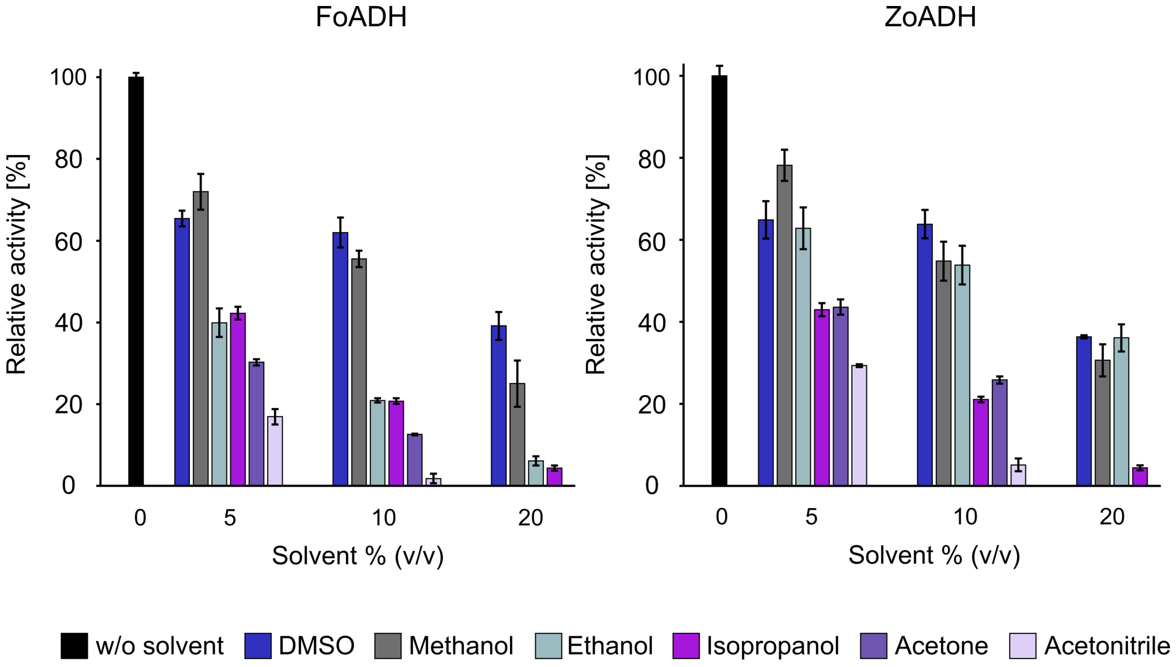

Fig. S8 Influence of water-miscible solvents on enzyme activity. The oxidation reaction was conducted in the presence of 5, 10, and 20% (v/v) solvent and compared with a control containing no additional solvent, which corresponds to the maximum relative activity (100%). The reaction was performed in 50 mM NaPi buffer at 25 °C. The final enzyme concentration was 0.1 mg mL^{-1},10 mM benzyl alcohol was employed as substrate and the reaction was started by adding 0.5 mM NAD^{+}. All measurements were performed as triplicates, the mean and the standard deviation is given.

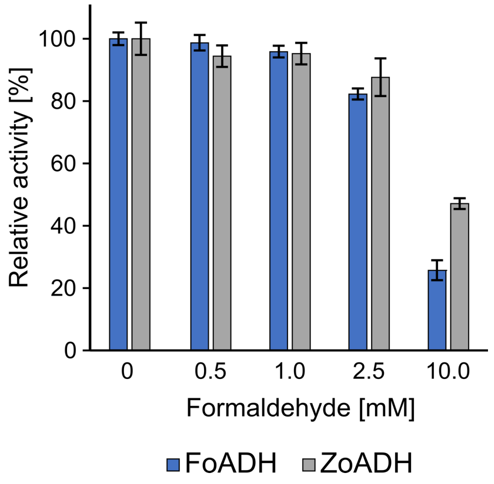

Fig. S9 Influence of formaldehyde on enzyme activity. The enzymes were incubated at a concentration of 1 mg mL^{-1} with different concentrations of formaldehyde varying from 0 to 50 mM for 1 hour at RT prior activity measurement to evaluate the effect of formaldehyde on enzyme activity. The measurement of the sample without formaldehyde corresponds to the maximum relative activity (100%). No activity was observed for both enzymes in the presence of 50 mM formaldehyde. The reaction was performed in 50 mM HEPES buffer pH 8.5 at 25 °C. The final enzyme concentration was 0.1 mg mL^{-1},10 mM benzyl alcohol was employed as substrate and the reaction was started by adding 0.5 mM NAD^+. All measurements were performed as triplicates, the mean and the standard deviation is given.

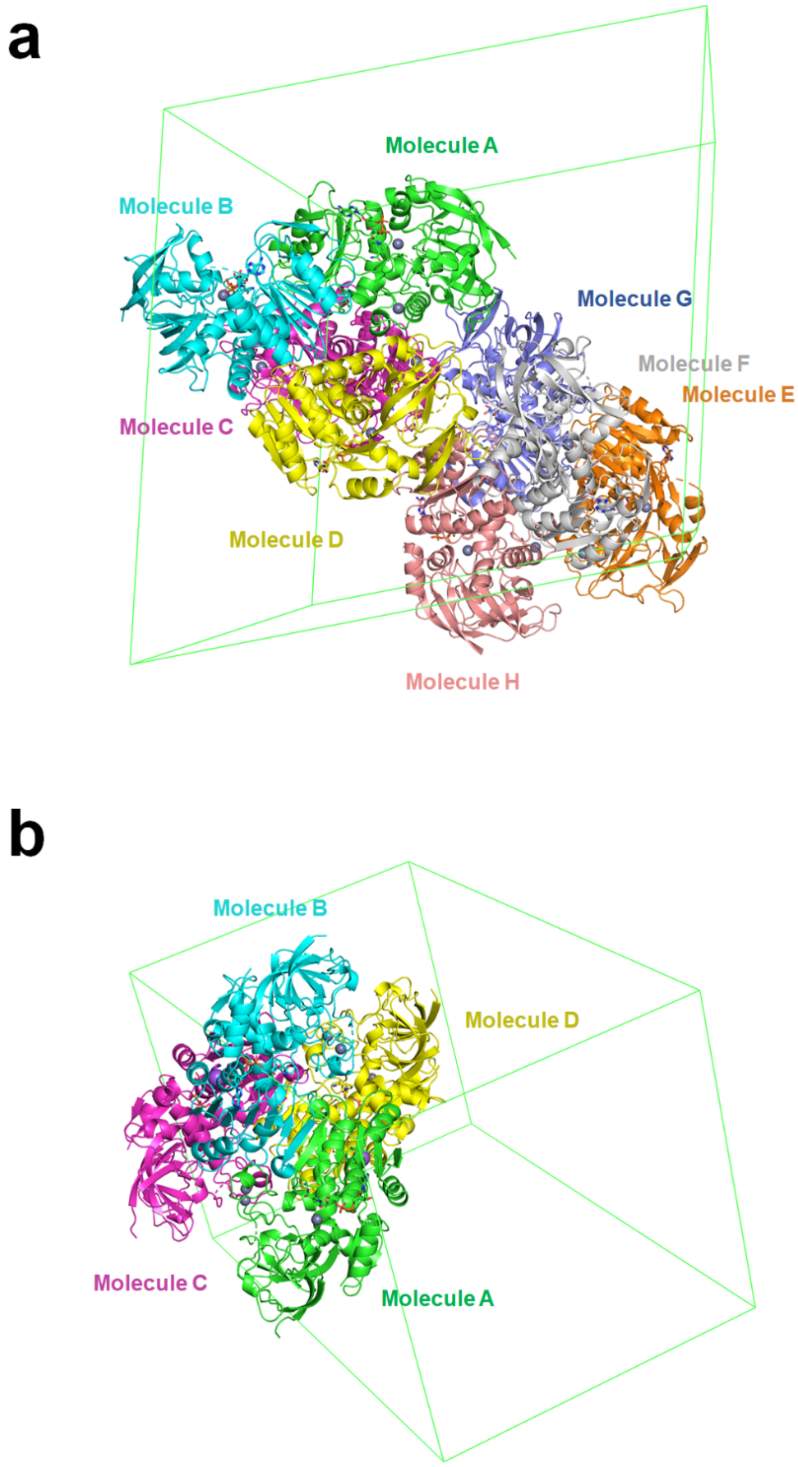

Fig. S10 Asymmetric units of both ADHs. **a**) Eight FoADH molecules and **b**) four ZoADH molecules in asymmetric unit.

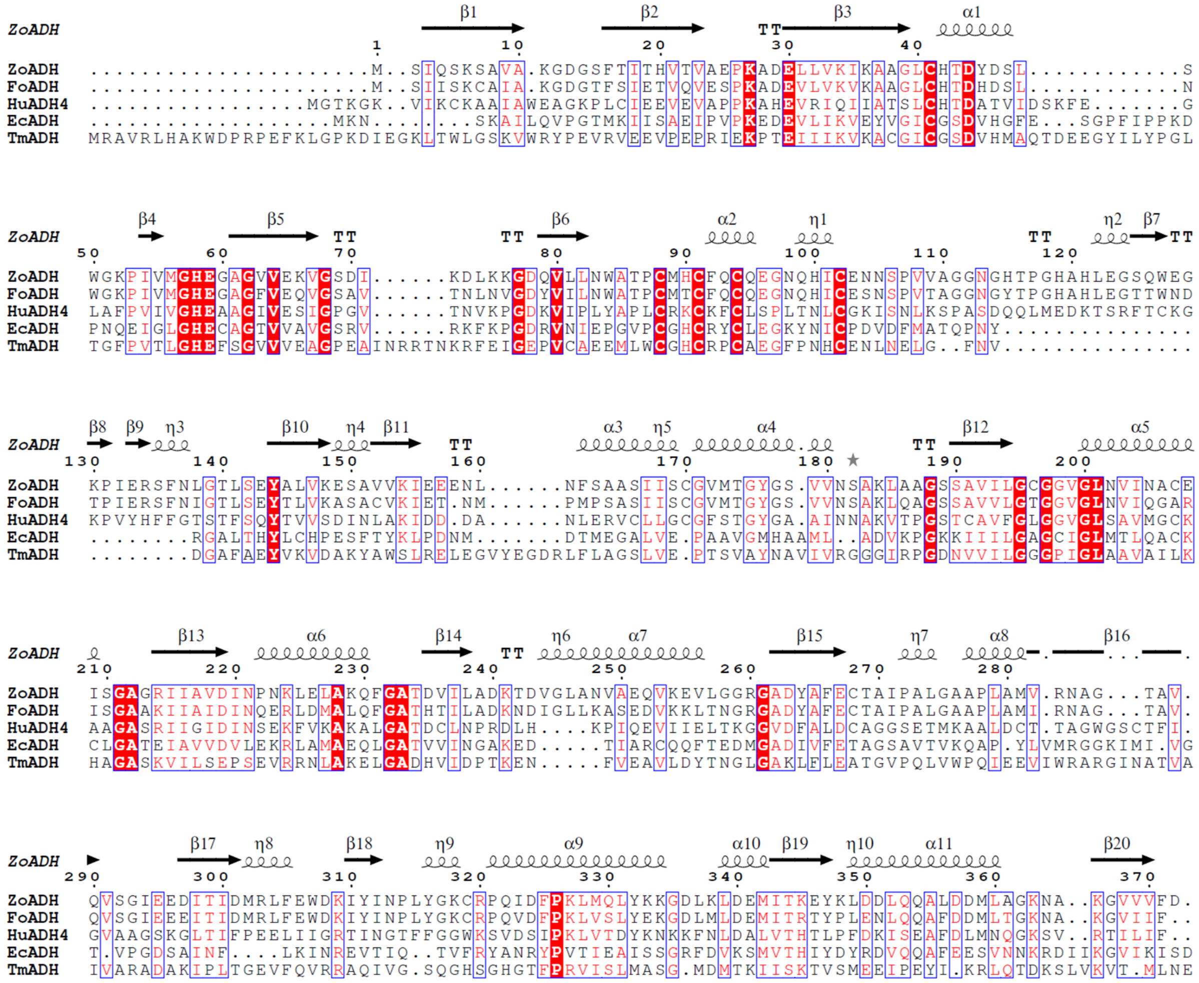

Fig. S11 Structure-based amino acid alignment of ZoADH (UniProt: G0L712) and FoADH (T2KM87) with all-trans-retinol dehydrogenase ADH4 from *Homo sapiens* (P08319), uncharacterized zinc-type alcohol dehydrogenase-like protein YdjJ from *Escherichia coli* (P77280) and scyllo-inosose 3-dehydrogenase from *Thermotoga maritima* (Q9WYP3).

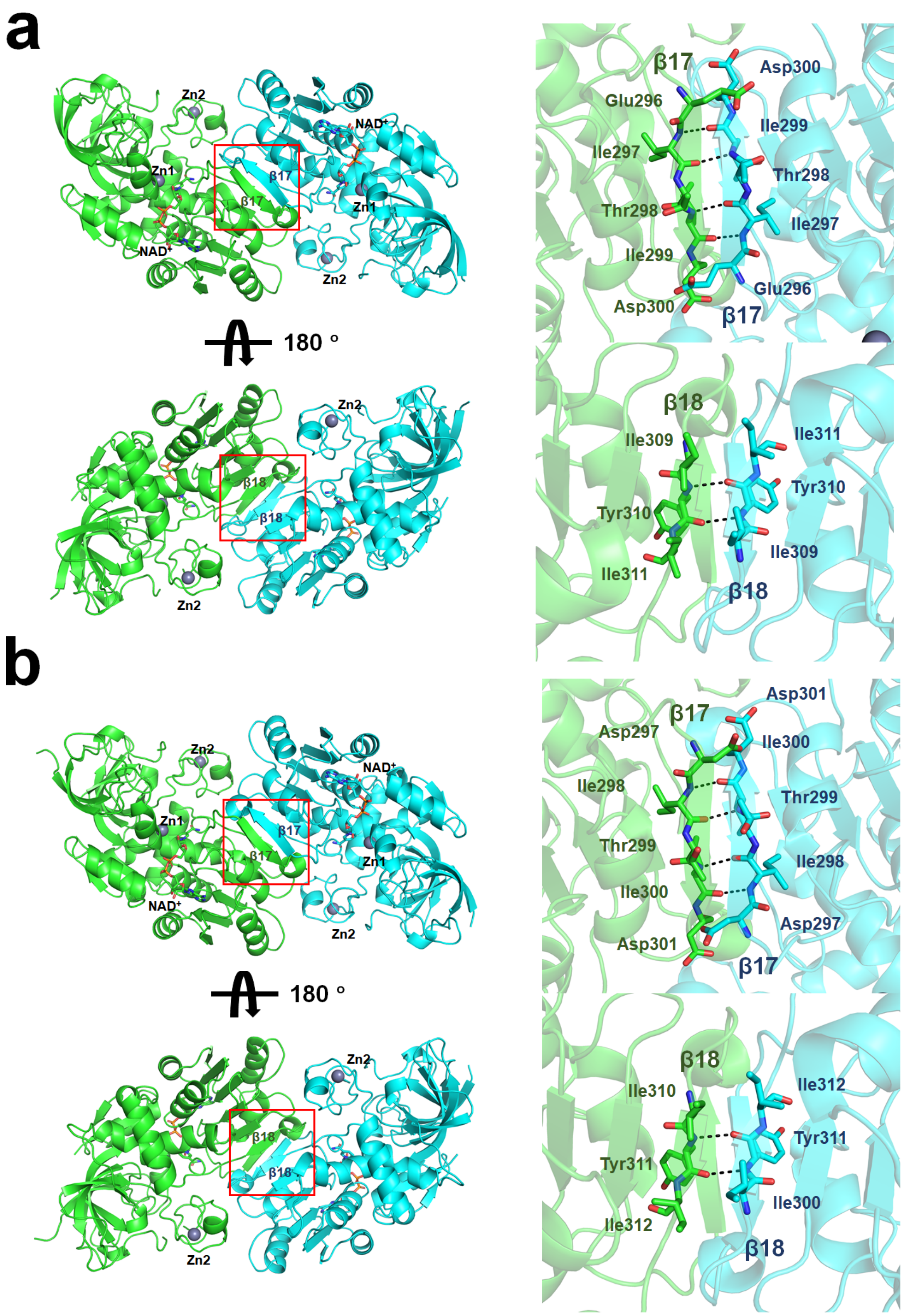

Fig. S12 Dimeric interface of **a**) FoADH and **b**) ZoADH.

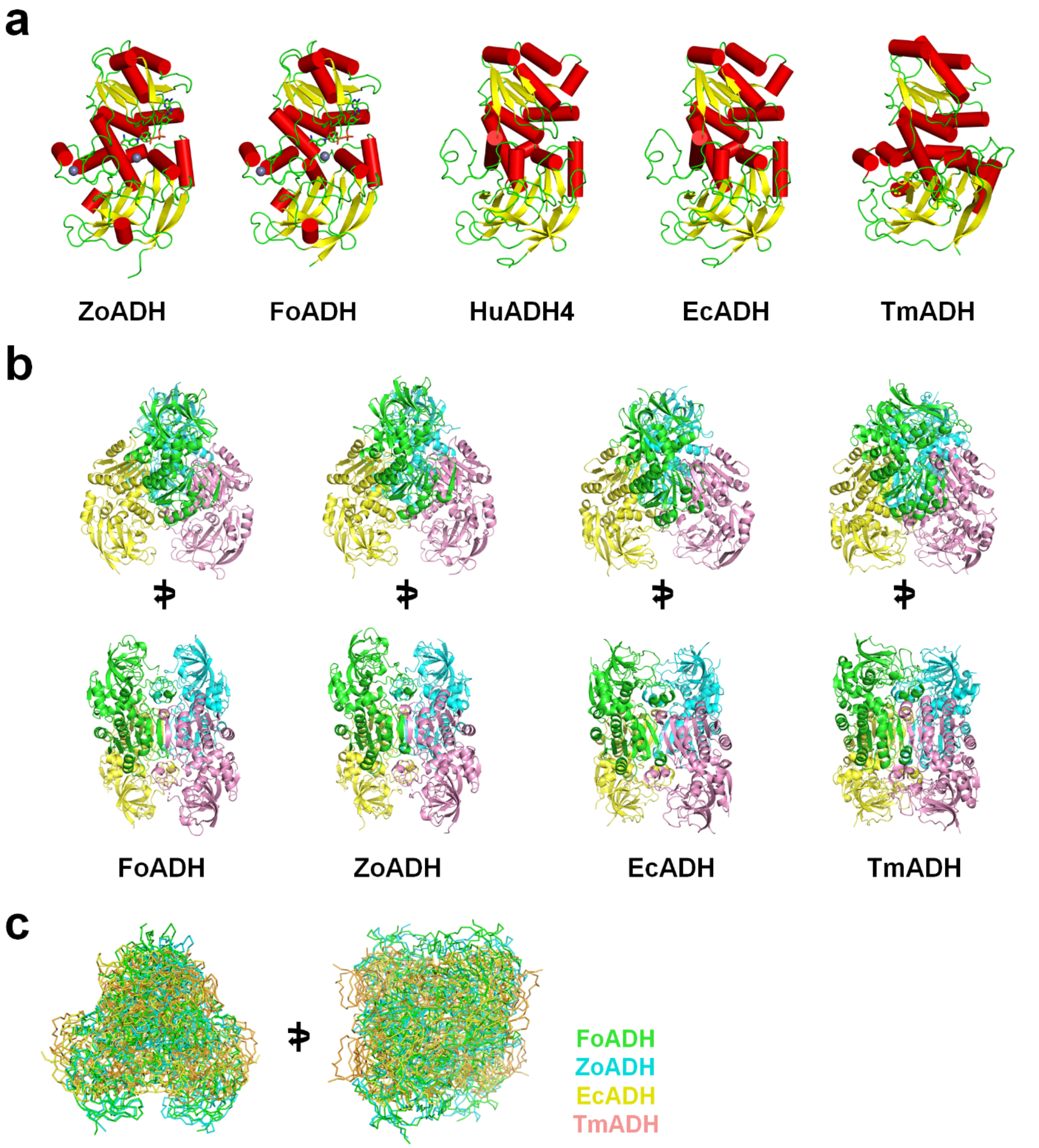

Fig. S13 Structural comparison of ZoADH and FoADH with structural homologs. **a**) Comparison of monomeric ZoADH and FoADH with structural homolog all-trans-retinol dehydrogenase ADH4 from *Homo sapiens* (HuADH4, PDB code 3COS), uncharacterized zinc-type alcohol dehydrogenase-like protein YdjJ from *Escherichia coli* (EcADH, 5vm2) and scyllo-inosose 3-dehydrogenase from *Thermotoga maritima* (TmADH, 3IP1). **b**) Comparison and **c**) superimposition of tetrameric assembly of FoADH, ZoADH, EcADH and TmADH.

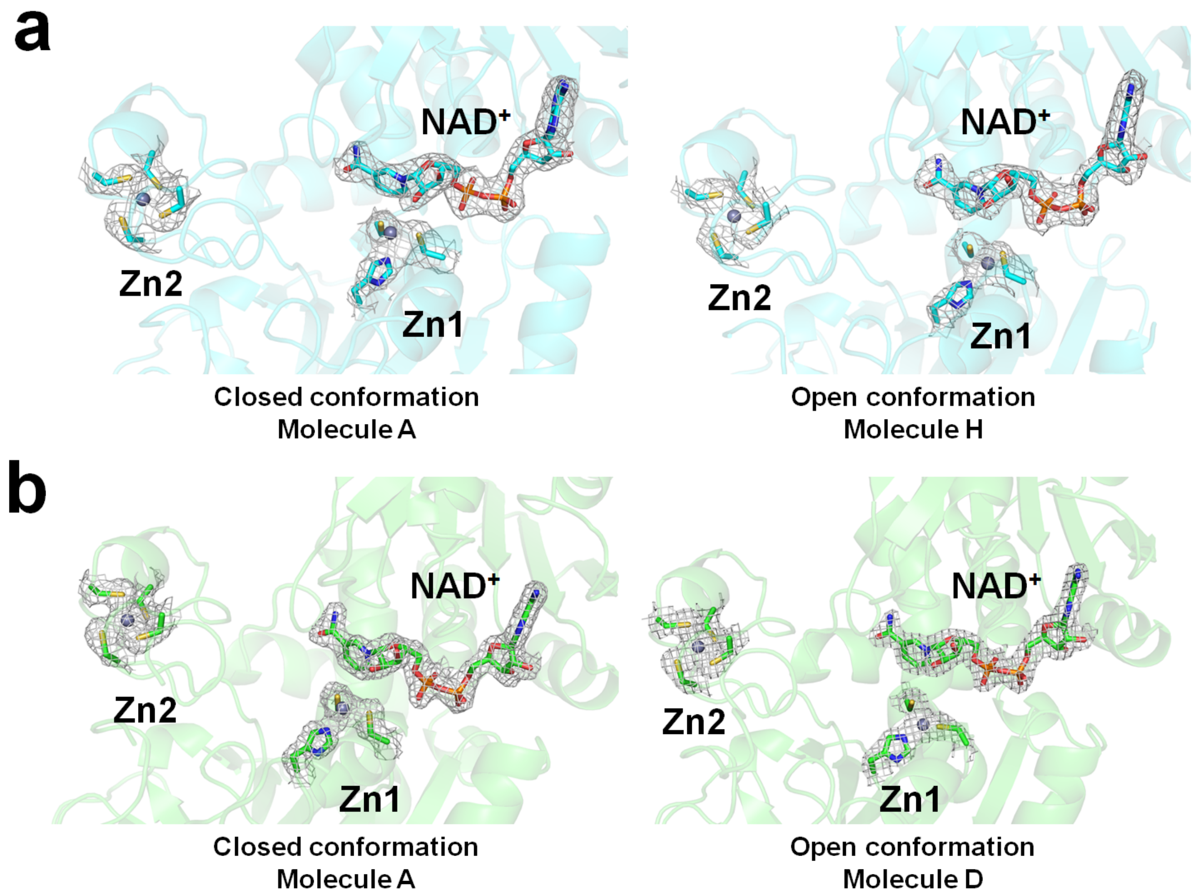

Fig. S14 2mFo-DFc (blue mesh, 1σ) electron density map NAD^{+} and Zn binding of **a**) FoADH and **b**) ZoADH.

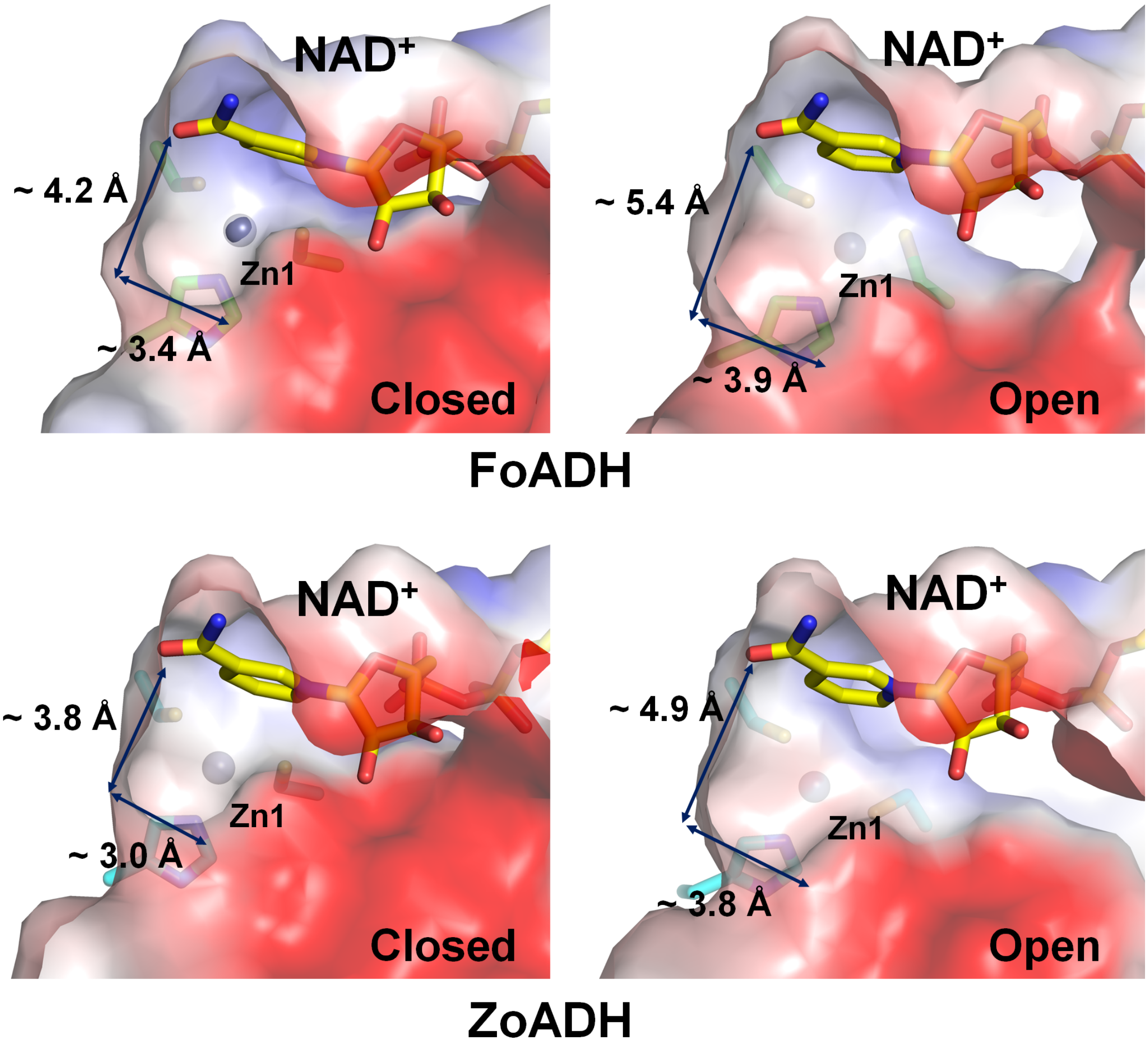

191

192 **Fig. S15** Substrate binding pocket of FoADH and ZoADH.

193

Tables

Table S1 Primers used for knockout experiments.

Primers	Sequence and Description
OFT0040	5' TTTTTTGTCGACTGCGTAAAGAACCTGAGATTACCG 3'; forward primer used in construction of pFT13; SalI site underlined
OFT0041	5' TAGGCCGCTTCTTTTTTAGGATCG 3'; reverse primer used in construction of pFT12; BamHI site downstream in amplified fragment
OFT0042	5' TTTTTTTCTAGAGCTACTGCACTCTTTGATTGAATAG 3'; reverse primer used in construction of pFT13; XbaI site underlined
OFT0043	5' TTTTTTTCTAGAGTTGGCCGGTAAAAATGCAAAAGG 3'; forward primer used in construction of pFT12; XbaI site underlined
OFT0044	5' ACACCCTTTTGTCCGTTGATGCG 3'; forward primer to confirm deletion of *zgal_4674*
OFT0045	5' AAATCTTCCAAGTCGGCCATTGG 3'; reverse primer to confirm deletion of *zgal_4674*

Table S2 Data collection and refinement statistics**.**

**Data collection**	**FoADH**	**ZoADH**
Beamline	11C beamline, PLS-II	11C beamline, PLS-II
Wavelength (Å)	0.9794	0.9794
Space group	$P2_1$	$P2_12_12_1$
Unit cell		
a, b, c (Å)	98.49, 157.20, 98.57	90.57, 92.67, 154.19
α, β, γ (°)	90.00, 103.50, 90.00	90.00, 90.00, 90.00
Resolution	50.0-2.50 (2.54-2.50)	50.0-2.10 (2.14-2.10)
Total reflections	98086 (4819)	76289 (3735)
Redundancy	5.7 (4.6)	12.6 (11.3)
Completeness (%)	97.5 (95.9)	99.9 (100.0)
Mean I/sigma(I)	12.79 (1.93)	15.00 (1.94)
CC1/2	0.976 (0.751)	0.989 (0.734)
CC*	0.994 (0.926)	0.997 (0.920)
**Refinement**		
Resolution	48.19-2.50	49.58-2.10
R_{work}	0.225	0.1612
R_{free}	0.278	0.2047
R.M.S. Deviation		
Bonds (Å)	0.010	0.009
Angles (°)	1.424	1.200
Average B-factor ($Å^2$)		
Protein	51.20	33.45
Ligand	49.89	29.13
Water	45.75	36.81
Ramachandran (%)		
Favored	94.53	96.10
Allowed	4.77	3.69
Outliers	0.70	0.21

Statistics for the highest-resolution shell are shown in parentheses.

Table S3 The ADHs lacked activity for various sugar substrates. For the measurement an incubation temperature of 40 °C was used. Sugars were used at a final substrate concentration of 30 mM. The reactions were performed in the 50 mM NaPi pH 8.5 for the oxidation and 50 mM succinate pH 6.5 for the reduction reaction. The final enzyme concentration was 0.1 mg mL^{-1}. The reaction was initialized by the addition of 0.5 mM NAD^+ or NADH.

Sugar substrates	
6-*O*-methyl-D-galactose	D-Sucrose
D-Galactose	D-Trehalose
D-Glucose	3,6-Anhydro-D-galactose
D-Fructose	L-Rhamnose
D-Xylose	L-Arabinose
D-Mannose	

Table S4 Superimposition of monomer structures of FoADH and ZoADH

		ZoADH			
	**Chain**	**A**	**B**	**C**	**D**
FoADH	**A**	0.562	0.391	0.350	0.621
	**B**	0.420	0.396	0.357	0.672
	**C**	0.402	0.383	0.396	0.543
	**D**	0.625	0.691	0.772	0.498
	**E**	0.419	0.399	0.347	0.650
	**F**	0.497	0.454	0.487	0.472
	**G**	0.435	0.405	0.381	0.647
	**H**	0.546	0.597	0.651	0.459

* Numbers indicate r.m.s. deviation values.

* Chain marked in blue have an open conformation between cofactor and catalytic domain.

Table S5 Superimposition of monomeric structures of FoADH.

Chain	A	B	C	D	E	F	G	H
**A**		0.256	0.310	0.579	0.226	0.460	0.263	0.562
**B**	0.256		0.353	0.626	0.259	0.568	0.248	0.610
**C**	0.310	0.353		0.494	0.320	0.413	0.353	0.457
**D**	0.579	0.626	0.494		0.587	0.336	0.608	0.276
**E**	0.226	0.259	0.320	0.587		0.500	0.264	0.574
**F**	0.460	0.568	0.413	0.336	0.500		0.550	0.350
**G**	0.263	0.248	0.353	0.608	0.264	0.550		0.576
**H**	0.562	0.610	0.457	0.276	0.574	0.350	0.576	

* Numbers indicate r.m.s. deviation values.

* Chain marked in blue have an open conformation between cofactor and catalytic domain.

Table S6 Superimposition of monomeric structures of ZoADH.

Chain	A	B	C	D
**A**		0.198	0.226	0.314
**B**	0.198		0.207	0.418
**C**	0.226	0.207		0.471
**D**	0.314	0.418	0.471	

* Numbers indicate r.m.s. deviation values.

* Chain marked in blue have an open conformation between cofactor and catalytic domain.

Table S7 Hydrogen bonds and salt bridges on the interface of FoADH.

**Molecule A-B**					
**Hydrogen bonds**			**Salt bridges**		
Molecule A	Dist. [Å]	Molecule B	Molecule A	Dist. [Å]	Molecule B
Asn283 [O]	3.37	Gln93 [NE2]	Asp307 [OD1]	3.78	His100 NE2]
Asp307 [OD2]	2.51	His100 [NE2]	Asp307 [OD2]	2.51	His100 [NE2]
Glu103 [OE2]	3.48	Arg259 [NE]	Glu103 [OE1]	3.50	Arg259 [NE]
Glu103 [OE1]	3.01	Arg259 [NH2]	Glu103 [OE2]	3.48	Arg259 [NE]
Glu305 [OE2]	3.32	Ile293 [N]	Glu103 [OE1]	3.01	Arg259 [NH2]
Ile299 [O]	2.90	Ile297 [N]	His100 [NE2]	3.56	Asp307 [OD1]
Ile297 [O]	2.86	Ile299 [N]	His100 [NE2]	2.37	Asp307 [OD2]
Glu295 [O]	2.95	Met301 [N]	Arg259[NE]	3.90	Glu103 [OE1]
Ile293 [O]	3.55	Arg302 [N]			
Asn312 [O]	2.94	Asp307 [N]			
Tyr310 [O]	2.80	Tyr310 [N]			
Glu305 [OE2]	3.78	Asn312[ND2]			
Asp307 [OD1]	2.87	Leu314 [N]			
Gln93 [NE2]	2.87	Asn283 [O]			
His100 [NE2]	2.37	Asp307 [OD2]			
Ile293 [N]	3.54	Glu305 [OE2]			
Ile297 [N]	3.06	Ile299 [O]			
Ile299 [N]	2.88	Ile297 [O]			
Met301 [N]	3.34	Glu295 [O]			
Arg302 [N]	3.45	Ile293 [O]			
Trp306 [N]	3.30	Asn312 [O]			
Asp307 [N]	3.02	Asn312 [O]			
Tyr310 [N]	2.83	Tyr310 [O]			
Leu314 [N]	3.27	Asp307 [OD1]			
**Molecule C-D**					
**Hydrogen bonds**			**Salt bridges**		
Molecule C	Dist. [Å]	Molecule D	Molecule C	Dist. [Å]	Molecule D
Asn283 [O]	3.03	Gln93 [NE2]	Asp307 [OD1]	3.18	His100 [NE2]
Glu103 [OE1]	3.18	Arg259 [NH2]	Asp307 [OD2]	2.89	His100 [NE2]
Glu305 [OE2]	3.26	Ile293 [N]	Glu103 [OE1]	3.18	Arg259 [NH2]
Ile299 [O]	3.02	Ile297 [N]	Glu103 [OE2]	3.79	Arg259 [NH2]
Ile297 [O]	2.84	Ile299 [N]	His100 [NE2]	3.65	Asp307 [OD1]
Glu295 [O]	3.04	Met301 [N]	His100 [NE2]	2.74	Asp307 [OD2]
Ile293 [O]	3.37	Arg302 [N]	Arg259 [NE]	3.65	Glu103 [OE2]
Asn312 [OD1]	3.41	Trp306 [N]	Arg259 [NE]	3.17	Glu103 [OE1]

Asn312 [O]	3.03	Trp306 [N]	Arg259[NH2]	3.72	Glu103 [OE1]
Asn312 [O]	3.23	Asp307 [N]			
Tyr310 [O]	2.94	Tyr310 [N]			
Asp307 [OD2]	3.44	Leu314 [N]			
Gln93 [NE2]	2.71	Asn283 [O]			
Arg259 [NE]	3.17	Glu103 [OE1]			
Ile297 [N]	3.19	Ile299 [O]			
Ile299 [N]	2.82	Ile297 [O]			
Met301 [N]	3.15	Glu295 [O]			
Arg302 [N]	3.74	Ile293 [O]			
Trp306 [N]	3.22	Asn312 [O]			
Asp307 [N]	3.03	Asn312 [O]			
Tyr310 [N]	2.88	Tyr310 [O]			
Leu314 [N]	3.86	Asp307 [OD2]			
**Molecule A-C**					
**Hydrogen bonds**					
Molecule A	Dist. [Å]	Molecule C			
Gln185 [NE2]	3.81	VAL 179 [O]			
Gln185 [NE2]	3.37	Asn180 [OD1]			
**Molecule B-D**					
**Hydrogen bonds**					
Molecule B	Dist. [Å]	Molecule D			
Ala211[O]	2.93	LYS 333 [NZ]			
**Molecule E-G**					
**Hydrogen bonds**			**Salt bridges**		
Molecule E	Dist. [Å]	Molecule G	Molecule E	Dist. [Å]	Molecule G
Asn283 [O]	2.93	Gln93 [NE2]	Asp307 [OD1]	3.16	His100 [NE2]
Glu103 [OE1]	2.37	Arg259 [NH2]	Asp307 [OD2]	3.28	His100 [NE2]
Glu305 [OE2]	3.65	Ile293 [N]	Glu103 [OE1]	2.37	Arg259 [NH2]
Ile299 [O]	3.12	Ile297 [N]	Glu103 [OE2]	3.57	Arg259 [NH2]
Ile297 [O]	3.02	Ile299 [N]	His100 [NE2]	2.52	Asp307 [OD2]
Glu295 [O]	3.01	Met301 [N]	Arg259 [NE]	3.44	Glu103 [OE1]
Ile293 [O]	3.53	Arg302 [N]	Arg259 [NE]	3.11	Glu103 [OE2]
Asn312 [OD1]	3.54	Trp306 [N]	LYS 317 [NZ]	3.46	Asp307 [OD1]
Asn312 [O]	3.13	Trp306 [N]	LYS 317 [NZ]	3.31	Asp307 [OD2]
Asn312 [O]	2.95	Asp307 [N]			
Tyr310 [O]	3.08	Tyr310 [N]			
Asp307 [OD2]	3.44	Leu314 [N]			
Gln93 [NE2]	2.95	Asn283 [O]			

His100 [NE2]	2.52	Asp307 [OD2]			
Arg259 [NE]	3.11	Glu103 [OE2]			
Arg282 [NH2]	3.79	Gln93 [OE1]			
Ile293 [N]	3.66	Glu305 [OE2]			
Ile297 [N]	3.36	Ile299 [O]			
Ile299 [N]	3.00	Ile297 [O]			
Met301 [N]	2.71	Glu295 [O]			
Arg302 [N]	3.72	Ile293 [O]			
Trp306 [N]	3.03	Asn312 [O]			
Asp307 [N]	2.92	Asn312 [O]			
Tyr310 [N]	2.83	Tyr310 [O]			
Leu314 [N]	2.87	Asp307 [OD1]			
**Molecule F-H**					
**Hydrogen bonds**			**Salt bridges**		
Molecule F	Dist. [Å]	Molecule H	Molecule F	Dist. [Å]	Molecule H
Asn283 [O]	3.00	Gln93 [NE2]	Asp307 [OD1]	3.87	His100 [NE2]
Asp307 [OD2]	2.37	His100 [NE2]	Asp307 [OD2]	2.37	His100 [NE2]
Glu103 [OE2]	3.15	Arg259 [NE]	Glu103 [OE1]	3.91	Arg259 [NE]
Glu103 [OE1]	2.64	Arg259[NH1]	Glu103 [OE2]	3.15	Arg259 [NE]
Glu305 [OE2]	3.45	Ile293 [N]	Glu103 [OE1]	2.64	Arg259[NH1]
Ile299 [O]	2.88	Ile297 [N]	Glu103 [OE2]	3.35	Arg259[NH1]
Ile297 [O]	3.50	THR 298[OG1]	His100 [NE2]	2.56	Asp307 [OD2]
Ile297 [O]	2.77	Ile299 [N]	Arg259 [NE]	3.05	Glu103 [OE2]
Glu295 [O]	3.17	Met301 [N]	Arg259 [NH2]	3.07	Glu103 [OE1]
Ile293 [O]	3.43	Arg302 [N]	Arg259 [NH2]	3.47	Glu103 [OE2]
Glu294 [O]	3.53	Arg302[NH1]			
Asn312 [O]	3.22	Trp306 [N]			
Asn312 [O]	3.09	Asp307 [N]			
Tyr310 [O]	2.77	Tyr310 [N]			
Asp307 [OD1]	2.85	Leu314 [N]			
Gln93 [NE2]	3.10	Asn283 [O]			
His100 [NE2]	2.56	Asp307 [OD2]			
Arg259 [NE]	3.05	Glu103 [OE2]			
Arg259 [NH2]	3.07	Glu103 [OE1]			
Arg282 [NH2]	3.70	Gln93 [OE1]			
Ile293 [N]	3.90	Glu305 [OE2]			
Ile297 [N]	3.03	Ile299 [O]			
Ile299 [N]	2.80	Ile297 [O]			
Met301 [N]	3.20	Glu295 [O]			

Arg302 [N]	3.51	Ile293 [O]
Trp306 [N]	3.22	Asn312 [O]
Asp307 [N]	2.90	Asn312 [O]
Tyr310 [N]	2.84	Tyr310 [O]
Leu314 [N]	2.97	Asp307 [OD1]
**Molecule G-H**		
**Hydrogen bonds**		
Molecule G	Dist. [Å]	Molecule H
Ala211 [O]	3.21	LYS 333 [NZ]
Gly187[N]	3.87	Tyr175 [OH]
**Molecule E-F**		
**Hydrogen bonds**		
Molecule E	Dist. [Å]	Molecule F
Tyr175 [OH]	3.86	Gly187 [N]
Ala211 [O]	3.12	LYS 333 [NZ]
Gly187[N]	3.81	Tyr175 [OH]

Table S8 Hydrogen bonds and salt bridges on the interface of ZoADH.

**Molecule A-B**					
**Hydrogen bonds**			**Salt bridges**		
Molecule A	Dist. [Å]	Molecule B	Molecule A	Dist. [Å]	Molecule B
Asn284 [O]	3.13	Gln93 [NE2]	Asp308 [OD2]	2.48	His100 [NE2]
Asp308 [OD2]	2.48	His100 [NE2]	Asp308 [OD1]	3.50	His100 [NE2]
Glu103 [OE1]	2.80	Arg260 [NE]	Glu103 [OE1]	2.80	Arg260 [NE]
Glu103 [OE2]	3.03	Arg260 [NH2]	Glu103 [OE2]	3.77	Arg260 [NE]
Glu306 [OE2]	3.64	Ile294 [N]	Glu103 [OE1]	3.56	Arg260 [NH2]
Ile300 [O]	3.04	Ile298 [N]	Glu103 [OE2]	3.03	Arg260 [NH2]
THR 299[OG1]	3.67	THR 299[OG1]	Glu 96 [OE2]	3.99	Arg283 [NH2]
Ile298 [O]	2.85	Ile300 [N]	His100 [NE2]	2.35	Asp308 [OD2]
Glu296 [O]	2.97	Met302 [N]	His100 [NE2]	3.76	Asp308 [OD1]
Ile294 [O]	3.40	Arg303 [N]	Arg260 [NE]	2.86	Glu103 [OE1]
Asn313 [O]	2.98	Asp308 [N]	Arg260 [NE]	3.67	Glu103 [OE2]
Tyr311 [O]	2.89	Tyr311 [N]	Arg260 [NH2]	3.63	Glu103 [OE1]
Asp308 [OD1]	2.90	Leu315 [N]	Arg260 [NH2]	2.90	Glu103 [OE2]
Gln93 [NE2]	2.97	Asn284 [O]	LYS 318 [NZ]	3.80	Asp308 [OD2]
His100 [NE2]	2.35	Asp308 [OD2]	LYS 318 [NZ]	3.76	Asp308 [OD1]
Arg260 [NE]	2.86	Glu103 [OE1]			
Arg260 [NH2]	2.90	Glu103 [OE2]			
Ile294 [N]	3.35	Glu306 [OE2]			
Ile298 [N]	3.00	Ile300 [O]			
Ile300 [N]	2.87	Ile298 [O]			
Met302 [N]	2.87	Glu296 [O]			
Arg303 [N]	3.37	Ile294 [O]			
Asp308 [N]	2.97	Asn313 [O]			
Tyr311 [N]	2.93	Tyr311 [O]			
Leu315 [N]	2.90	Asp308 [OD1]			

**Molecule C-D**					
**Hydrogen bonds**			**Salt bridges**		
Molecule C	Dist. [Å]	Molecule D	Molecule C	Dist. [Å]	Molecule D
Asn284 [O]	3.06	Gln93 [NE2]	Asp308 [OD1]	3.56	His100 [NE2]
Asp308 [OD2]	2.45	His100 [NE2]	Asp308 [OD2]	2.45	His100 [NE2]
Glu103 [OE1]	2.60	Arg260 [NE]	Glu103 [OE1]	2.60	Arg260 [NE]
Glu103 [OE2]	2.77	Arg260 [NH2]	Glu103 [OE2]	3.66	Arg260 [NE]
Glu306 [OE2]	3.49	Ile294 [N]	Glu103 [OE1]	3.34	Arg260 [NH2]
Ile300 [O]	3.07	Ile298 [N]	Glu103 [OE2]	2.77	Arg260 [NH2]
THR 299[OG1]	3.63	THR 299[OG1]	His100 [NE2]	3.75	Asp308 [OD1]

Ile298 [O]	2.84	Ile300 [N]	His100 [NE2]	2.19	Asp308 [OD2]
Glu296 [O]	3.02	Met302 [N]	Arg260[NH1]	3.42	Glu103 [OE2]
Ile294 [O]	3.45	Arg303 [N]	Arg260[NH1]	3.37	Glu103 [OE1]
Asn313 [O]	2.93	Asp308 [N]	Arg260 [NH2]	2.65	Glu103 [OE2]
Tyr311 [O]	2.88	Tyr311 [N]	Arg260 [NH2]	3.88	Glu103 [OE1]
Asp308 [OD1]	2.98	Leu315 [N]			
Gln93 [NE2]	2.98	Asn284 [O]			
His100 [NE2]	2.19	Asp308 [OD2]			
Arg260[NH1]	3.37	Glu103 [OE1]			
Arg260 [NH2]	2.65	Glu103 [OE2]			
Ile294 [N]	3.47	Glu306 [OE2]			
Ile298 [N]	3.00	Ile300 [O]			
Ile300 [N]	2.92	Ile298 [O]			
Met302 [N]	3.07	Glu296 [O]			
Arg303 [N]	3.45	Ile294 [O]			
Asp308 [N]	2.95	Asn313 [O]			
Tyr311 [N]	2.86	Tyr311 [O]			
Leu315 [N]	2.87	Asp308 [OD1]			
**Molecule A-D**					
**Hydrogen bonds**			**Salt bridges**		
Molecule A	Dist. [Å]	Molecule D	Molecule A	Dist. [Å]	Molecule D
Gly213 [O]	2.78	Gln330 [NE2]	Glu208 [OE1]	3.85	LYS 334 [NZ]
Ala212 [O]	2.82	LYS 334 [NZ]	Glu208 [OE2]	3.03	LYS 334 [NZ]
Glu208 [OE2]	3.03	LYS 334 [NZ]	LYS 334 [NZ]	2.95	Glu208 [OE1]
Gln330 [NE2]	2.95	Gly213 [O]			
LYS 334 [NZ]	2.95	Ala212 [O]			
LYS 334 [NZ]	2.95	Glu208 [OE1]			
**Molecule B-C**					
**Hydrogen bonds**			**Salt bridges**		
Molecule B	Dist. [Å]	Molecule C	Molecule B	Dist. [Å]	Molecule C
Gln330 [NE2]	3.16	Gly213 [O]	LYS 334 [NZ]	2.82	Glu208 [OE1]
Gly213 [O]	3.15	Gln330 [NE2]	LYS 334 [NZ]	3.58	Glu208 [OE2]
Glu208 [OE1]	2.82	LYS 334 [NZ]			

References

Akal AL, Karan R, Hohl A, Alam I, Vogler M, Grötzinger SW, Eppinger J, Rueping M (2019) A polyextremophilic alcohol dehydrogenase from the Atlantis II Deep Red Sea brine pool. FEBS Open Bio 9:194–205. https://doi.org/10.1002/2211-5463.12557

Barbeyron T, L'Haridon S, Corre E, Kloareg B, Potin P (2001) *Zobellia galactanovorans* gen. nov., sp. nov., a marine species of Flavobacteriaceae isolated from a red alga, and classification of [*Cytophaga*] *uliginosa* (ZoBell and Upham 1944) Reichenbach 1989 as *Zobellia uliginosa* gen. nov., comb. nov. Int J Syst Evol Microbiol 51:985–997. https://doi.org/10.1099/00207713-51-3-985

Bubb WA, Berthon HA, Kuchel PW (1995) Tris Buffer Reactivity with Low-Molecular-Weight Aldehyds: NME Characterization of the reactions of glyceraldehyde 3-phosphate. Bioorg Chem 23:119–130. https://doi.org/10.1006/bioo.1995.1010

Nedashkovskaya OI, Kim SB, Vancanneyt M, Snauwaert C, Lysenko AM, Rohde M, Frolova GM, Zhukova N V., Mikhailov V V., Bae KS, Oh HW, Swings J (2006) *Formosa agariphila* sp. nov., a budding bacterium of the family Flavobacteriaceae isolated from marine environments, and emended description of the genus *Formosa*. Int J Syst Evol Microbiol 56:161–167. https://doi.org/10.1099/ijs.0.63875-0

Smith KW, Johnson SL (1975) Borate inhibition of yeast alcohol dehydrogenase. Biochemistry 34:560–565. https://doi.org/10.1021/bi00648a016

Trivić S, Leskovac V, Zeremski J, Stančić B, Anderson BM (1998) Influence of Tris(hydroxymethyl)aminomethane on kinetic mechanism of yeast alcohol dehydrogenase. J Enzyme Inhib 13:57–68. https://doi.org/10.3109/14756369809035827

Uthoff S, Steinbüchel A (2012) Purification and Characterization of an NAD^+-Dependent XylB-like aryl alcohol dehydrogenase identified in *Acinetobacter baylyi* ADP1. Appl Environ Microbiol 78:8743–8752. https://doi.org/10.1128/AEM.02224-12

Ying X, Wang Y, Xiong B, Wu T, Xie L, Yu M, Wang Z (2014) Characterization of an allylic/benzyl alcohol dehydrogenase from *Yokenella sp*. strain WZY002, an organism potentially useful for the synthesis of α,β-unsaturated alcohols from allylic aldehydes and ketones. Appl Environ Microbiol 80:2399–2409. https://doi.org/10.1128/AEM.03980-13

Zhang L, Jiang D, Li Y, Wu L, Liu Q, Dong K, Oger P (2021) Characterization of a novel type III alcohol dehydrogenase from *Thermococcus barophilus* Ch5. Int J Biol Macromol 171:491–501. https://doi.org/10.1016/j.ijbiomac.2020.12.197
